# Co-Produce, Co-Design, Co-Create, or Co-Construct—Who Does It and How Is It Done in Chronic Disease Prevention? A Scoping Review

**DOI:** 10.3390/healthcare10040647

**Published:** 2022-03-30

**Authors:** Bronwyn McGill, Lucy Corbett, Anne C. Grunseit, Michelle Irving, Blythe J. O’Hara

**Affiliations:** 1 Prevention Research Collaboration, Charles Perkins Centre, Sydney School of Public Health, The University of Sydney, Camperdown, NSW 2006, Australia; lucy.corbett@sydney.edu.au (L.C.); anne.grunseit@sydney.edu.au (A.C.G.); blythe.ohara@sydney.edu.au (B.J.O.); 2 The Australian Prevention Partnership Centre, 235 Jones Street, Ultimo, NSW 2007, Australia; michelle.irving@sydney.edu.au; 3 Menzies Centre for Health Policy, School of Public Health, Faculty of Medicine and Health, University of Sydney, Camperdown, NSW 2006, Australia

**Keywords:** co-produce, co-design, co-construct, health promotion, chronic disease prevention

## Abstract

Co-production in health literature has increased in recent years. Despite mounting interest, numerous terms are used to describe co-production. There is confusion regarding its use in health promotion and little evidence and guidance for using co-produced chronic disease prevention interventions in the general population. We conducted a scoping review to examine the research literature using co-production to develop and evaluate chronic disease prevention programs. We searched four electronic databases for articles using co-production for health behaviour change in smoking, physical activity, diet, and/or weight management. In 71 articles that reported using co-production, co-design, co-create, co-develop, and co-construct, these terms were used interchangeably to refer to a participatory process involving researchers, stakeholders, and end users of interventions. Overall, studies used co-production as a formative research process, including focus groups and interviews. Co-produced health promotion interventions were generally not well described or robustly evaluated, and the literature did not show whether co-produced interventions achieved better outcomes than those that were not. Uniform agreement on the meanings of these words would avoid confusion about their use, facilitating the development of a co-production framework for health promotion interventions. Doing so would allow practitioners and researchers to develop a shared understanding of the co-production process and how best to evaluate co-produced interventions.

## 1. Introduction

In Australia, as in many other high-income countries, chronic diseases place a significant and persistent burden on the community, with the social and economic consequences having a detrimental effect on an individual’s quality of life [1]. Much of the burden caused by chronic disease is preventable, with modifiable risk factors including tobacco use, overweight and obesity, physical inactivity, and unhealthy dietary behaviours [2]. Governments are often the lead agency for the development and the implementation of health promotion strategies and programs to prevent health behaviour-related chronic disease at the population level, as well as research into their effectiveness as a way of promoting healthy choices, preventing disease, and keeping people out of hospital [3,4]. ‘Co-production’ is a mechanism whereby ‘stakeholders’ (identified as including end users or intervention target audience, health researchers, academics, policy and practice partners, decision makers, and funding representatives) can collaborate, generate relevant knowledge, and apply it to practice [5,6,7]. It involves key stakeholders in the development of health-related interventions based on the premise that involving the target audience or intermediaries in the design and implementation is likely to have a positive impact on the effectiveness of the service or program [8].

The number of publications that identify the use of co-production in health literature has risen markedly in recent years. While it has been suggested that the growth has been approximately 25% per year from 2004 to 2019 [9], a keyword search for ‘health’ and ‘co-production’ using the Web of Science database indicates that the increase appears to be even steeper over the past ten years. However, little is known about how co-production facilitates the development of effective programs. Clarke et al. [10] found that evaluations of the outcomes of co-produced interventions designed to improve the quality of acute health care services lacked rigour, particularly when assessing clinical and service outcomes and cost-effectiveness. It appears that co-production is difficult to evaluate and cannot be evaluated by the standard evidence hierarchy in the evidence-based practice movement [11]. While some evaluation frameworks exist, effectiveness is mostly demonstrated through the use of case studies [12]. Despite mounting interest, there is limited evidence and guidance for co-produced chronic disease prevention interventions in the general population [13], and to our knowledge, there are no reviews.

Another factor that may hinder the advancement of the field is the heterogeneity of the terms used to describe such a process in the health setting (e.g., co-produce, co-design, co-create) [14], a problem also reported in the public administration setting [15]. That literature has sought to clarify the concept by considering who is involved in co-production (and at what level: individual, group, or collective), what occurs in co-production, and when it occurs [16]. Similarly, untangling the confusion around co-production in health and chronic disease prevention settings is important. There is a need, and interest, to identify where the literature could be enhanced using a shared understanding of what co-production is (and is not), what other terms might be used to mean the same thing, who the key players in co-production are, and what about co-production might make an intervention effective [17,18].

The purpose of this study, therefore, was to undertake a systematic scoping review to determine the size and extent of available research literature on the use of co-production in the development and evaluation of primary research studies of health behaviour change programs for the prevention of chronic disease. In particular, we sought to answer (a) how co-production is used in the development and evaluation of chronic disease prevention programs, (b) who is usually involved in the co-production of the development and evaluation of chronic disease prevention programs, and (c) whether and how the literature reports on the evaluation of co-produced chronic disease prevention programs.

## 2. Materials and Methods

We conducted a scoping review to identify the nature and extent of co-produced interventions addressing chronic disease prevention [19]. A scoping review was chosen because its purpose is to identify the types of evidence in a research field, clarify key concepts, explore how research is conducted on a topic, and identify gaps in the literature [20]. The methodology was guided by an established framework for scoping studies [21,22]. The Preferred Reporting Items for Systematic reviews and Meta-analyses extension for Scoping Reviews (PRISMA-ScR) checklist was used to ensure that this scoping review achieved quality standards of practice and reproducibility [23].

The development of the search strategy was iterative. First, we conducted a preliminary search of the literature, which included the term ‘co-production’ to identify other terms related to co-production that were commonly used in the literature [6,14]. These terms were: co-design, co-create, co-construct, partnership, and collaboration. A further literature search was conducted to identify a sample of articles using ‘partnership’ or ‘collaboration’ in the development and evaluation of chronic disease prevention programs. Two authors (B.M. and B.O’H.) screened the full text of these papers to determine how partnership (n = 16) and collaboration (n = 12) were used in these studies. We found articles using the terms ‘collaboration’ or ‘partnership’ did so to loosely refer to intersectoral (including academic, government, and financial) relationships. The studies screened did not define or describe what was meant by partnership or collaboration in sufficient detail in relation to the development or evaluation of programs with a chronic disease prevention focus for these terms to be included in this study. This observation is supported by Johnston and Finegood [24], who acknowledge the ambiguous and interchangeable use of the term partnership with other terms including collaboration. Therefore, ‘partnership’ and ‘collaboration’ were excluded from the final search strategy as they were concepts considered broader than co-production.

We systematically searched electronic databases for peer-reviewed literature (Medline via Ovid, PsycINFO via Ovid, Cinahl, Scopus, and PubMed) for articles using a co-production, co-design, co-creation, and co-construction approach to achieve a lifestyle-related health behaviour change [6,14]. The search was conducted in May 2020 and updated in March 2021. Search terms included a combination of medical subject headings (MeSH) terms and keywords as outlined in Table S1. Reference lists of all included studies and relevant interventions known to the authors were searched for additional studies. We included English, peer-reviewed articles with studies reporting protocols and/or outcomes of interventions (primary and secondary prevention) using a co-production, co-design, co-creation, and co-construction approach in any health promotion setting. The outcomes of interest were chronic disease prevention-related behaviours outcomes such as smoking, physical activity, diet, and/or weight management. The populations of interest included individuals, communities, or populations at risk of developing health behaviour-related chronic disease. We did not limit studies by research design or publication year. Studies of interventions with a clinical orientation (e.g., those targeting service delivery, rehabilitation, or medication adherence) were excluded.

Search results were combined in EndNote X9.3.3, and duplicate references were removed. Three authors (B.M., B.O’H., and L.C.) independently scanned titles and abstracts to determine inclusion eligibility. The full text of eligible papers was independently reviewed by B.M., B.O’H., and L.C. according to the pre-determined inclusion criteria outlined above, with discrepancies resolved by discussion. Study characteristics extracted included how study authors defined the ‘co-words’ used, who was involved in the co-production, the process and impact measures to evaluate interventions using co-production, and the sections of the paper where co-words were mentioned. Data were further tabulated according to the purpose of the study, the prevention focus, the target population, and the collaborative technique used in relation to co-production (Table S2).

## 3. Results

### 3.1. Study Selection

**Figure 1:**
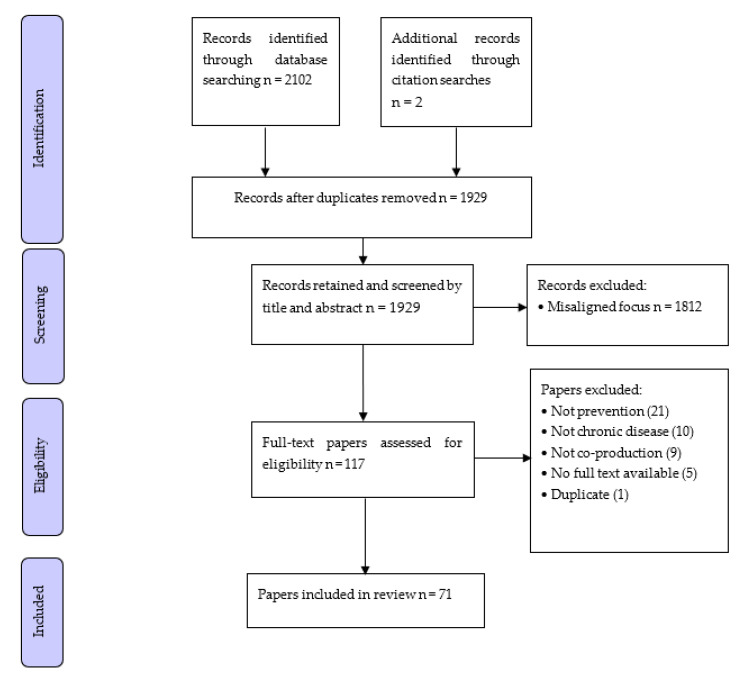
Flowchart of study selection and exclusion.

Database searches identified 589 publications, and an additional two publications were identified by citation searching. Following duplicate removal and title and abstract screening, 117 full-text articles were reviewed for inclusion eligibility, resulting in the inclusion of 71 articles (64 unique studies and 7 reviews) (Figure 1).

### 3.2. Study Characteristics

#### 3.2.1. Publication Country

Articles that address co-production were from a range of countries: the U.K. (England [25,26,27,28,29,30,31,32,33], Scotland [34,35,36] and Wales [13,37]), the European Union [38] (The Netherlands [39,40,41,42,43,44,45,46], Belgium [47,48], Denmark [49,50], Ireland [51], Italy [52,53], Estonia [54], Sweden [55], Greece [56], France [57] and Spain, Italy and U.K. [58,59]), Australia [60,61,62,63,64,65,66,67,68,69,70,71,72], USA [73,74,75,76,77], Canada [78,79], New Zealand [80,81], Brazil [82] and Lebanon [83]. The studies included in review articles had an global focus, and authors were from Australia [84,85,86], New Zealand [87], the U.K. [88,89] and Germany, Switzerland, Australia, The Netherlands, USA and Canada [90]. The review included seven protocol papers [32,34,46,48,68,77,81], seven review papers [84,85,86,87,88,89,90] and the remaining described intervention development and/or the impact of the intervention.

#### 3.2.2. Prevention Focus

The *prevention focus* of articles included health promotion and healthy lifestyle [40,41,42,43,65,73,81,84,85,87,90,91,92], health policy and chronic disease prevention [32,62,66,88], obesity prevention [33,34,35,49,52,58,59,61,77,80,89,93], physical activity [25,30,37,38,39,47,48,51,53,54,79,82,83], physical inactivity [44], sedentary behaviour or sitting [27,31,64], healthy eating or dietary behaviour change [26,46,57,68,69,71,74,75,78], smoking cessation or prevention [13,45,56,60,70] and alcohol abuse prevention [50,63,94] (Table 1). The target populations in terms of general age-groups included children [33,34,35,37,39,42,43,46,77,83,93], adolescents [40,41,47,51,52,58,61,84,85,86,91,92,94] or both [25,54,65], families of preschool children [49], mothers of infants and children [73,75], fathers and their children [48], young adults [45,68,78], adults [28,29,53], adults with intellectual disability [88], and older adults [30,36,38,44,55,79]. More specifically, food outlet managers or consumers [26,69,72], Aboriginal or First Nation people and communities [13,60,63,67,70,71,74,76,80,81], ethnic [66] or socioeconomically disadvantaged communities [32,33,57,73], and desk-based workers [27,31,64] and commercial stakeholders [56] were also the focus.

#### 3.2.3. ‘Co-Word’

Articles used five different ‘co-‘ words: co-design, co-create, co-produce, co-construct and co-develop. While these words appeared throughout the text of the articles, they were used at least once in the abstracts of 65 articles and in the discussion and conclusions of 57 articles. Five articles used the word co-design in only the abstract [26,60,66,74,77]. As a single term, co-design (n = 25, 34.7%) [26,53,55,58,59,60,61,63,64,65,66,67,71,74,76,77,78,79,80,81,84,86,87,91,93], co-create (n = 17, 24%) [27,33,36,38,39,40,41,45,46,47,48,50,51,56,73,75,85] and co-produce (n = 8, 11.1%) [13,29,31,34,35,37,89,90] were used most frequently. Co-construct (n = 2, 2.8%) [44,57] and co-develop (n = 1, 1.4%) [25] were the least prevalent. In a quarter of the articles (n = 18, 25%) two or more ‘co-‘ words were used interchangeably in the same paper, most frequently including co-design and co-create [42,49,69,72,82,92], co-design and co-develop [30,70], and co-create and co-design [54,68]. For consistency, from this point the words ‘co-production’ or ‘co-produce’ will be used to refer to all ‘co-‘ words (co-design, co-create, co-develop and co-construct).

### 3.3. The Operationalisation of Co-Production in the Development and Evaluation of Chronic Disease Prevention Interventions

Co-design was reported in 39 articles (including 4 reviews) [25,26,30,32,42,49,52,53,54,55,58,59,60,61,63,64,65,66,67,68,69,70,71,72,74,76,77,78,79,80,81,82,83,84,86,87,88,91,92,93]. Of these, seven explicitly defined co-design as it was used in the development of a chronic disease prevention intervention [53,63,69,72,82,84,87], 28 described how they used co-design but did not define it explicitly [25,26,32,42,49,52,54,55,58,59,60,61,65,66,70,71,74,76,77,78,79,80,81,83,88,91,92,93] and four did not specify what was meant by co-design [30,64,67,86]. All articles that defined co-design, plus an additional article with a co-creation focus [68], identified that co-design aligned with a participatory design approach, in which end users or stakeholders of intervention are engaged in the research process. As one of the papers stated, the process of co-design is illustrated as a ‘golden thread’ running through all stages of public health research [84], enabling the contributions of end users to be incorporated from intervention development and testing to implementation and dissemination [63,82,87]. In particular, the aim is to empower stakeholders as part of the design process by recognising their expertise in their own experiences [53,69]. Intervention co-design was described in these studies as an iterative and creative collaboration or partnership between end users and relevant stakeholders and intervention designers [53,63,84,87]. While not all articles explicitly defined what they meant by co-design, collaboration between multiple stakeholders as part of the co-design process was described in the methods in the majority of papers reporting co-design [25,53,54,55,58,60,61,65,66,74,79,80,81,82,83,84,87,92]. In terms of stakeholders involved, in the studies we identified intervention development occurred in consultation with the community or industry [60,65,66,70,71,74,76] and end users [42,43,55,58,61,79,91,92].

Co-creation was included in 23 articles (22 unique studies and 1 review) [27,36,38,39,40,41,42,43,45,46,47,48,49,50,51,56,62,68,72,73,75,85,94]. Six included a definition of co-creation [38,45,48,68,72,85], 12 described how they incorporated co-creation [27,36,39,40,41,47,49,50,51,56,73,75], and despite using the term within their paper, five did not clearly outline what was meant by co-creation in the study [42,43,46,62,94]. In those papers that did define it, co-creation was defined as an active process between people with shared goals but different expertise and skills, by which stakeholders were enabled to be directly involved in the generation of an intervention or solution [38,45,48,68,72,85]. There were similarities in how co-creation was defined to the definitions of co-design described above. Namely, co-creation is described as having developed from participatory design [85] and the collaborative engagement of all stakeholders, including end users, throughout the process of developing and implementing an intervention [38,48,68,72,85]. Two articles described the inclusion of co-creation in the development of behaviour change interventions as (a) reducing barriers to change [56] and (b) providing insights into motivation for change [51].

Co-production was reported in 16 articles (13 unique studies and 3 reviews) [13,25,28,29,31,32,33,34,35,37,52,62,88,89,90,94]. Two studies provided an explicit definition of co-production [33,37], eight described the process by which co-production was used to develop or evaluated their intervention [13,28,29,32,34,35,52,90], and six did not provide details on what they meant by co-production [25,31,62,88,89,94]. Similar to co-design and co-creation, co-production was defined as involving the target audience in the design and implementation of an intervention [37]. The process of co-production, according to these articles, incorporated the implementation, stakeholder, and participant contexts into the intervention development, implementation, and evaluation [32]. As with co-design and co-creation, co-production was described as using participatory approaches to involve participants and stakeholders in an equal and reciprocal relationship [35,52] for the iterative development of an intervention [13,28,29]. The authors felt that such a process gave rise to services that meaningfully met the needs of individuals and communities, and represented an engaging process to achieve behaviour change among end users [33].

Co-construction was used in three unique studies [44,57,62]. None defined co-construction but described the process as involving collaboration with stakeholders and end users through all stages of intervention development and evaluation [44,57,62].

A variety of co-production techniques were described, but there was no clear pattern of use according to which ‘co’-word was used (Table 2). Group sessions, focus groups and discussions were used in 31 unique studies (34 articles) [13,26,28,29,32,33,34,35,37,39,40,41,42,43,44,46,47,48,51,53,59,62,67,73,74,76,77,78,80,81,83,91,92,93], workshops with stakeholders were used in 20 studies [25,30,33,36,38,49,50,55,57,58,61,63,68,69,72,79,82,89,92,94], interviews in 16 studies [13,27,30,32,35,37,44,48,50,57,65,68,73,76,77,93] and surveys or questionnaires in 10 studies [28,29,32,51,52,61,65,78,91,93]. Other techniques used included social media [45,68], observations in combination with a workshop or focus group, [13,63] an engagement event [26], a school visit [54], yarning circle or storytelling [62,70,94], and virtual simulation [50].

### 3.4. Those Involved in the Co-Production of Chronic Disease Prevention Interventions

Co-production was used to different extents depending on whether the intervention was in a development or implementation phase or whether the study was reporting on the evaluation of the intervention. We found 59 studies that reported using co-production in the development of an intervention, in a description akin to formative research [13,25,26,27,28,29,30,31,33,34,35,36,37,38,39,40,41,42,43,44,45,46,47,48,49,50,51,53,54,55,57,58,59,60,61,62,63,64,65,66,67,68,69,70,72,73,74,75,76,78,79,80,81,82,83,91,92,93,94]. In this development phase, we found academics (n = 55) [13,25,26,27,28,29,30,31,33,34,35,36,37,38,39,40,41,42,43,44,45,46,47,48,49,50,51,53,54,55,57,58,59,60,61,62,63,64,65,66,68,73,74,75,76,78,79,80,81,82,83,91,92,93,94], representatives from the target population (n = 54) [13,25,27,28,29,30,31,33,34,35,36,37,38,39,40,41,42,43,44,45,46,47,48,49,50,51,53,54,55,57,58,59,60,61,62,63,66,67,68,69,70,72,73,74,76,78,80,81,82,83,91,92,93,94], intervention designers/advisers (n = 23) [13,25,28,29,34,37,38,44,46,49,50,60,61,62,65,68,70,73,74,76,83,92,93], implementers of interventions (n = 22) [13,25,26,28,29,33,35,38,40,41,55,60,64,65,66,67,70,75,76,78,79,80] and policy makers (n = 6) [37,40,41,42,43,76].

Seven studies reported the use of co-production practices throughout the implementation phase of the intervention [52,54,56,71,76,80,83] involving academics (n = 4) [54,76,80,83], representatives from the target population (n = 3) [54,76,83], intervention designers/advisers (n = 2) [76,83], implementers (n = 2) [76,80] and policy makers [76]. The design of the intervention evaluation was reported in 14 studies [28,29,42,43,47,57,60,66,67,68,75,76,80,83] with academics as contributors in all of these studies. The evaluation was designed and conducted by academics alone in just two [66,68] out of these 14 studies despite multiple stakeholders being involved in the development of the intervention itself. When the evaluation was co-produced, representatives of the target population (n = 9) [28,29,42,43,47,57,75,76,83], intervention designers/advisers (n = 4) [28,29,76,83], implementers (n = 7) [28,29,60,67,75,76,80] and policy makers (n = 3) [42,43,76] worked alongside academics.

### 3.5. Evaluation of Chronic Disease Prevention Interventions Developed Using Co-Production

Ten studies included mention of the acceptability [26,31,51,60,92] and feasibility [26,27,31,36,47,51,58,60,73] of implementing a co-produced intervention. Among these studies, there was no consistency in the way acceptability and feasibility were measured. Most authors concluded that a co-produced intervention was feasible because the views of the target audience were able to be incorporated into a revised intervention. Similarly, acceptability was determined by implication because of the acceptability of the target audience’s involvement or as measured post-intervention development through questionnaires or qualitative interviews. Three protocol papers included plans to undertake a process evaluation, with some consideration given to issues of implementation of a co-produced intervention [32,35,77].

Eighteen studies reported on the evaluation of their intervention in terms of process evaluation (n = 5) [43,44,45,50,92], impact evaluation (n = 9) [47,57,65,66,67,69,71,78,79] and both process and impact evaluation (n = 4) [29,52,56,76]. These studies did not include an analysis of whether the use of co-production afforded any implementation or outcome advantage and, as such, did not report on the impact that co-production had on the associated implementation or outcomes of the program. A further two-thirds of the papers (65.3%, n = 47) included in the review limited their scope to describing only the techniques used for undertaking co-production, with no evaluation results reported of any kind.

## 4. Discussion

This scoping review found 71 articles that reported using co-production when developing a chronic disease prevention intervention or program, with the majority published in the last three years. Our findings highlight that different ‘co’- words were used interchangeably within and across many studies, and little attention was paid to whether there were any differences (subtle or otherwise) in their intended use and meaning. The ‘co’- words used included co-produce, co-design, co-create, co-develop, and co-construct, either singly or in combination. Although we initially focused on co-production, co-design and co-creation were more commonly used in practice in the selected primary studies. Occasionally, a ‘co’- word was used only in the abstract, perhaps as a way of drawing attention to the article, but the body text included no further exploration of the term.

Across the different terms, in the studies we reviewed, ‘co-‘ words were used to describe a process of engaging with the target audience of end users or intermediaries (e.g., health promotion and health practitioners, etc.) [95] of an intervention in a participatory fashion [17]. There were no substantive differences in meaning between co-design, co-create, co-produce, and co-construct and how they were deployed in reporting on an intervention. There were also no notable differences in the methods used in co-production based on the ‘co-‘ word used by the study. Overall, co-production constituted a formative research process [96], including focus groups, interviews, and other methods of information collection. Again, the literature would benefit from clarity as to whether the different terms are or should be linked in some way to particular techniques.

Through our analysis, it became apparent that those who use a co-production method choose the relevant stakeholders to be involved in the design, implementation, or evaluation of a chronic disease prevention intervention. The most common co-production participants were academics and the target audience, followed by intervention designers, implementers, and policymakers. This finding is not unexpected given that the majority of studies in our review reported on co-production in terms of the development of an intervention rather than the implementation and evaluation. It is uncertain whether there would be a benefit in attempting to define the group(s) to be involved in co-production processes, as this may vary widely with the project and context. More important is to examine the impact of including different groups on the outcomes and implementation of an intervention.

As noted above and by other authors [18,96], our review confirmed the paucity of evidence that examines the impact or effectiveness of co-production processes in chronic disease prevention interventions. This is unsurprising given that most studies included in this review outline the co-production technique, and the few that reported on evaluations used a pre-post design in relation to the interventions’ target behaviours. A few studies noted that using a co-production method was acceptable and feasible because a) the studies had been effective at incorporating the views of the target audience in the design of the intervention and b) post design, the intervention had been used by the target audience almost as a proxy measure of acceptability. Future studies should formally evaluate the perceived acceptability and feasibility of co-produced interventions within target populations rather than relying on proxy measures. The review papers we included also reported that studies were more likely to report on feasibility and acceptability rather than impact of the co-production process on intervention outcomes, with Eyles et al. [87] concluding that “sufficiency of reporting was poor, and no study undertook a robust assessment of efficacy” (p 160). Future studies with robust evaluation designs are needed to evaluate the effectiveness of co-produced health promotion interventions so the impact of the co-production method can be determined.

Our findings suggest that there would be merit in developing conceptual or definitional guidance as to what these words mean or include in the chronic disease prevention setting and whether there are differences in meaning or whether they can be used interchangeably to describe the same process. Our review supports the notion that ‘co’ is suggestive of a co-operative, collaborative, or participatory design [97], as noted by Blomkamp [98], but it is not possible to suggest from our findings any definite differences in meaning between the various ‘co’-words. There may be merit in developing a framework that provides greater understanding of the distinctions between various terminology. This could be progressed by borrowing from the health services [99,100] and public administration co-production literature [14,15] which provides some guidance in defining the most used co-words by articulating their differences and then providing a hierarchy of meanings that can be used to guide co-production in chronic disease prevention. Our research also suggests that starting points could be: defining terms by those involved in the collaborative process [6] or using a staged approach to co-production as mapped against a program design cycle [6,13]. There is also an opportunity to explore how co-production does or does not align with community-based participatory research [101] or participatory action research [102], particularly in relation to where along the intervention design and evaluation continuum it fits and also which stakeholders it involves.

This review provides an initial step in overviewing a growing field of research that is ‘messy’. While research co-design in health has been included in previous reviews [103], our review is novel in its focus on the use of co-production in the development and evaluation of co-produced chronic disease prevention interventions that aim to change a lifestyle behaviour. A strength is the inclusion of all ‘co-‘ words used in publications and the broad view taken to recognise similarities or differences in their use.

A number of limitations that may affect how the findings are interpreted need to be acknowledged. Only studies published in English were included, potentially excluding relevant studies published in other languages. The search was limited to peer-reviewed literature and did not include a grey literature search. It is possible that policy statements and reports relevant to co-produced chronic disease prevention interventions could have contributed to the review findings. Future reviews should include studies in other languages as well as from the grey literature. The wide range of study designs and research methods are drawn from for this review limited the options available for drawing conclusions for defining ‘co’-words and suggesting frameworks appropriate to health promotion interventions [21]. We did not conduct a quality appraisal of the primary studies included, which is consistent with scoping review methodology [19,21] but leads to a broad range of included material. The advantage of considering the breadth of literature is that it provides a structured overview of the current use of co-production in health promotion and provides direction for the focus of future research.

## 5. Implications for Practice and Research

Our review suggests that, as with co-production more broadly, co-produced health promotion interventions that aim to prevent chronic disease are not well described or robustly evaluated. The public health literature does not currently provide insight into whether co-produced interventions achieve better outcomes than those that are not co-produced [104]. Co-production, co-design, co-creation, and co-develop seem to be used interchangeably to refer to a participatory or collaborative process involving researchers, stakeholders, and end users involved in the development or evaluation of such interventions. Uniform agreement on the meanings of these words would avoid confusion surrounding their use and facilitate the development of guidelines and/or a co-production framework specific to health promotion interventions. Doing so would allow researchers to develop a shared understanding of the co-production process and how best to evaluate co-produced interventions.

## Figures and Tables

**Table 1:** Processes used to facilitate input from stakeholders during co-production.

First Author, Year	Purpose of Study	Type of Co-Production	Prevention Focus	Target Population	Collaboration Technique
Carins 2021	Formative	Co-design, -create	Healthy eating	Supermarket consumers	Workshops
Hardt 2021	Formative	Co-design	Obesity prevention	Children	Survey, discussion groups, interviews
Mooses 2021	Formative	Co-create, -design	Physical activity	Children/adolescents (7–16 years)	Network building, school visits
Ochieng 2021	Formative	Co-create	Healthy weight	Children (ethnic minority)	Focus groups, workshops
Castro 2020	Formative	Co-design, -create	Physical activity	Low-income adults (40–90 years)	Focus groups
Champion 2020	Formative	Co-design	Lifestyle risk factors	Secondary school students	Survey, focus groups
Corr 2020	Formative, impact	Co-create	Physical activity	Adolescent girls (15–17 years)	Questionnaire, focus groups
D’Addario 2020	Formative	Co-design	Physical activity	Physically inactive adults	Focus groups
Daly-Smith 2020	Formative	Co-produce, -design, -develop	Physical activity	School-aged children/adolescents	Stakeholder workshops
Hidding 2020	Formative	Co-create	Physical activity	Children (9–12 years old)	Concept mapping, focus groups
Martin 2020	Formative	Co-design	Healthy weight	Adolescents (13–16 years)	Workshop, individual testing
Parder 2020	Formative	Co-create, -produce	Alcohol abuse prevention	Adolescents (13–15 years)	Workshops, storytelling
Peiris-Hohn 2020	Formative, process	Co-design, co-create	‘Health’ including PA	Adolescents (16+ years)	Group sessions, workshops
Lems 2020 Lems 2019	Formative Formative	Co-create	Health promotion	Adolescent girls (12–15 years) and boys (12–18 years)	Small group sessions
Anselma 2019 Anselma 2020	Formative Process	Co-design, -create Co-create, -develop	Healthy lifestyle	Children (9–12 years)	Group sessions
Fournier 2019	Formative, process	Co-construct	Physical inactivity	Older adults	Group sessions, interviews
Gillespie 2019	Formative	Co-produce	Obesity prevention	Primary school-aged children	Focus groups, interviews
Goffe 2019	Formative	Co-design	Food portion sizing	Food outlet owners/managers	Discussions, engagement event
Gould 2019	Formative	Co-design	Smoking cessation	Pregnant Indigenous women	Not specified
Hoeeg 2019	Formative	Co-design, -create	Obesity prevention	Families of preschool children	Workshops
Mammen 2019	Formative	Co-create	Health messages	Rural, low-income mothers	Focus groups, interviews
Mistura 2019	Formative, impact	Co-design	Food purchasing	First-year university students	Focus groups, surveys
Morgan 2019	Formative	Co-produce	Physical activity	Girls (9–11 years)	Focus groups, interviews
Ojo 2019	Formative	Co-create	Workplace sitting	Desk-based workers	Interviews
Partridge 2019	Formative	Co-design	Obesity prevention	Adolescents (13–18 years)	Workshop, survey
Santina 2019	Formative	Co-design, -develop	Physical activity	Children (10–12 years)	Group meetings
Buckley 2018 Buckley 2019	Formative Process, impact	Co-develop, -produce Co-produce	Physical activity	Adults with controlled lifestyle-related health issues	Group meetings, focus groups, survey
Guell 2018	Formative	Co-design, -develop	Physical activity	Older adults	Interviews, workshops
Street 2018	Formative	Co-construct, -create, -produce	Health policy	Aboriginal people	Deliberative forum, storyboard
Te Morenga 2018	Formative	Co-design	Obesity prevention	Maori people	Focus groups
Verbiest 2018	Protocol	Co-design	Healthy lifestyle behaviour	Adult Maori people	
Durl 2017	Formative	Co-design	Alcohol education	Adolescents (14–16 years)	Workshop, feedback, observations
Hawkins 2017	Formative	Co-produce	Smoking prevention	Adolescents (12–19 years)	Focus groups, interviews, observations
Janols 2017	Formative	Co-design	Health behaviour change	Older adults	Workshops
Leask 2017	Formative	Co-create	Sedentary behaviour	Older adults	Workshops
Verloigne 2017	Formative, impact	Co-create	Physical activity	Adolescent girls (16 years)	Groups
Yuan 2017	Formative	Co-create	Physical activity	Older adults	Workshops
Chau 2016	Formative	Co-design	Sedentary behaviour	Adult call-centre workers	Not specified
Nu 2016	Formative	Co-design	Dietary pattern change	Indigenous community	Working group
Rosso 2016	Formative, impact	Co-design	Health promotion (sport)	Children and youth	Interviews, surveys
Standoli 2016	Formative	Co-design	Obesity prevention	Adolescents	Focus groups
Isbell 2015	Formative	Co-create	Nutrition education	Women, infants, children	Strategic planning meetings
Mackenzie 2015	Formative	Co-produce	Sitting	University employees	Not specified
Vallentin-Holbech 2020	Process	Co-create	Alcohol consumption	Adolescents (15–18 years)	Workshops, interviews, virtual simulation
van den Heerik 2017	Process	Co-create	Smoking prevention	Youth (15–25 years)	Social media, linguistic analysis
Ahmed 2020	Impact, process	Co-design	Healthy eating	Indigenous tribal community	Focus group interviews
Bogomolova 2021	Impact	Co-design, -create	Healthy eating	Supermarket consumers	Workshops
Brimblecombe 2020	Impact	Co-design	Healthy eating	Remote Aboriginal communities	Working groups
Gallegos 2020	Impact	Co-design	Chronic disease	Ethnic communities	Not specified
De Rosis 2020	Impact, process	Co-produce, -design	Obesity prevention	Adolescents	Questionnaire (for evaluation)
Skerletopoulos 2020	Impact, process	Co-create	Smoking indoors	Citizens, commercial stakeholders	
Fehring 2019	Impact	Co-design	Water consumption	Remote Aboriginal communities	Group meetings
McKay 2018	Impact	Co-design	Physical activity	Older adults	Workshops
Perignon 2017	Impact, formative	Co-construct	Healthy eating	Socioeconomic disadvantage	Workshops, interviews
Beckerman-Hsu 2020	Protocol (process)	Co-design	Obesity prevention	Low-income preschool children	Focus groups, interviews
Bovill 2021	Protocol (formative)	Co-design, -develop	Smoking cessation	Pregnant Aboriginal women	Yarning circles, e-mail survey
Latomme 2021	Protocol (formative)	Co-create	Physical activity	Fathers and their children	Group sessions, interviews
Nahar 2020	Protocol (process)	Co-produce, -design	Cardiovascular prevention	Disadvantaged populations	Focus groups, questionnaires, interviews
Folkvord 2019	Protocol (formative)	Co-create	Fruit and vegetable intake	Children (7–13 years)	Focus groups
Lombard 2018	Protocol (formative)	Co-design, -create	Healthy eating	Young adults (18–24 years)	Social media, interviews, workshops
Gillespie 2019	Protocol (process)	Co-produce	Obesity prevention	Preschool-aged children	Group meetings
Review style papers					
Taggart 2021	Review	Co-produce	Obesity	Adults (intellectual disabilities)	Workshops
Ruan 2020	Review	Co-design	Health behaviours	Adolescents	Content analysis
Rutten 2019	Review, comment	Co-produce	Active lifestyles	Population-wide	Systems approach
Partridge 2018	Review	Co-design	Healthy lifestyle	Adolescents	Focus groups, interviews
Raeside 2018	Review	Co-create	Healthy behaviours	Adolescents	Focus groups, workshops
Taggart 2018	Review	Co-design, -develop, -produce	Type 2 diabetes prevention	Adults (intellectual disabilities)	Focus groups, interviews
Eyles 2016	Review	Co-design	Health behaviour change	Not limited by population	Not limited by collaborative technique

Note: Studies are organised by purpose of study, and within the broad purpose, they are organised alphabetically by year with the most recent first.

**Table 2:** Techniques used in co-produced interventions.

Technique	Co-Design	Co-Create	Co-Produce	Co-Construct	Combination	Total
	n (%)	n (%)	n (%)	n (%)	n (%)	N
Group session	12 (34)	10 (28.6)	4 (11.4)	1 (2.9)	8 (22.9)	35
Workshop	7 (35.0)	5 (25.0)	2 (10.0)		6 (30.0)	20
Interviews	4 (25.0)	4 (25.0)	3 (18.8)	2 (12.5)	3 (18.8)	16
Survey/questionnaire	5 (45.5)	2 (18.2)			4 (36.4)	11
Storytelling					3 (100.0)	3
Social media		1 (50.0)			1 (50.0)	2
Observation	1 (50.0)		1 (50.0)			2
Event	1 (100.0)					1
School visit					1 (100.0)	1
Virtual simulation		1 (100.0)				1

## Data Availability

Not applicable.

## Supplementary Materials

The following supporting information can be downloaded at: https://www.mdpi.com/article/10.3390/healthcare10040647/s1, Table S1: Search strategy; Table S2: Data extraction table.
