# Supplementary material for: Co-Produce, Co-Design, Co-Create, or Co-Construct—Who Does It and How Is It Done in Chronic Disease Prevention? A Scoping Review
Source: Healthcare (Basel). 2022 Mar 30;10(4):647. doi: 10.3390/healthcare10040647 (PMC9029027; doi:10.3390/healthcare10040647)
Supplement: Supplementary file 1 [file healthcare-10-00647-s001.zip › healthcare-1630024-Supplementary.pdf]

Table S1: Co-production search terms (Medline via OVID)

Search	Terms
S1	co-production.mp
S2	co-produce*
S3	co-design*.mp
S4	co-creation.mp
S5	co-create*
S6	co-construct*
S7	partnership
S8	collaborat*
**S9**	**s1 or s2 or s3 or s4 or s5 or s6 or s7 or s8**
S10	health promotion.mp or Health Promotion
S11	public health.mp or Public Health
S12	chronic disease.mp or Chronic disease
S13	Prevention.mp
**S14**	**s10 or s11 or s12 or s13**
S15	Lifestyle.mp or Life Style
S16	smok*.mp or Smoking Cessation or Smoking
S17	Tobacco.mp or Tobacco/ or Tobacco Smoking/ or "Tobacco Use Cessation"
S18	physical activity.mp or Exercise
S19	Nutrition.mp
S20	Diet.mp or Diet
S21	weight loss.mp or Weight Loss
S22	body mass index'.mp or Body Mass Index/ or Body Weight
S23	overweight* or Overweight or Obesity
S24	walk* or Walking
S25	step*
**S26**	**s15 or s16 or s17 or s18 or s19 or s20 or s21 or s22 or s23 or s24 or S25**
S27	**S9 and s14 and s26**
	**Limit to English**
	**s9 with Collaboration (not collaborat*) and s14 and s26**
	**S9 without partnership (with collaborat*) and s14 and s26**
	**S9 without partnership (with collaboration) and s14 and s26**
	**S9 without collaborat* or collaboration and s14 and s26**
	**S9 without collaborat* or collaboration or partnership and s14 and s26**
	**S9 and s14 and s26 (without** overweight* or Overweight or Obesity)
	**S9 and s14 and s26 (without** overweight* or Overweight or Obesity or walk* or step*)
	**s9 with Collaboration (not collaborat*) and s14 and s26 (without** overweight* or Overweight or Obesity)
	**s9 with Collaboration (not collaborat*) and s14 and s26 (without** overweight* or Overweight or Obesity or walk* or step*)

* The asterisk denotes a ‘wildcard symbol’ to broaden the search to include words starting with the same letter (e.g. past tense or plural of the same word).

Table S2: Co-production scoping review data extraction table

Reference / Country	Aim / Methodology	What is meant by co-production in the development and evaluation of chronic disease prevention interventions?	Who is involved in the co-production of the development and evaluation of chronic disease prevention interventions?	How effective (process and impact measures) are chronic disease prevention programs developed using co-production processes?	Where in the paper is co-production (or other 'co-word') mentioned?
Bovill et al., 2021[1]<br><br>Australia	To describe a culturally responsive research protocol to develop meaningful supports to empower Aboriginal and Torres Strait Islander mothers to quit smoking during pregnancy.<br><br>Co-designed research process, using Indigenous ethical values to inform the development of a pilot smoking cessation program.	Not defined. Co-design/co-develop included:<br><br>a) Aboriginal and Torres Strait Islander women's preference for, and current use within the community of non-pharmacological approaches to smoking cessation.<br>b) Health provider attitudes to non-pharmacological approaches and supports requested by Aboriginal and Torres Strait Islander women for smoking cessation.	Aboriginal and Torres Strait Islander women and health providers, project team and research governance committee.<br><br>Process is co-owned with urban and regional Aboriginal and Torres Strait Islander communities.	Protocol paper – effectiveness not assessed.	*Co-design, co-develop*<br><br>Abstract<br><br>Key words<br><br>Background<br><br>Discussion
Carins et al., 2021[2]<br><br>Australia	To explore the role of community in encouraging greater food well-being and allied behaviours.<br><br>A community-wide approach to co-design, using co-design workshops.	Co-creation defined as a collaborative process of development encouraging engagement and empowerment of people. It means shifting from approaches that develop programs 'for' people, to those that develop programs 'with' people.<br><br>Co creation includes: value co-discovery (exploring what is valued by consumers and other stakeholders), value co-design (developing, experiencing and responding to new program or service elements) and value co-delivery (combining efforts to bring program or service ideas to life).<br><br>Five co design workshops were used, including feedback on health-based strategies found in previous programs, creating and designing new program ideas, including how retail could contribute to improving consumer well-being.	Consumers (n=24) and supermarket staff (n=8) from an Australian consumer retail cooperative.	Described the co design process and analysis of the information collected through this process. Analyses revealed two significant insights:<br><br>(1) Confirmation and new empirical evidence aligned with previous conceptualisations of food well-being and other literature detailing the positive role of food practices in well-being. (2) Novel and unique ways to incorporate a community-wide approach to food wellbeing initiatives.	*Co-design, co-create*<br><br>Title<br><br>Abstract<br><br>Background<br><br>Methods<br><br>Discussion
Latomme et al., 2021[3]<br><br>Belgium	To describe the protocol of the Run Daddy Run intervention study, focusing on improving (co-)PA of	The co-creation approach was defined as a strong and active collaboration	Fathers and researchers involved in 'co-creation groups' to develop and test the intervention.	Protocol paper – effectiveness not assessed.	*Co-create*<br><br>Title

Reference / Country	Aim / Methodology	What is meant by co-production in the development and evaluation of chronic disease prevention interventions?	Who is involved in the co-production of the development and evaluation of chronic disease prevention interventions?	How effective (process and impact measures) are chronic disease prevention programs developed using co-production processes?	Where in the paper is co-production (or other 'co-word') mentioned?
	fathers and children, and the prospected outcomes.<br>Co-creation approach based on the Behaviour Change Wheel and using 'co-creation' groups.	between end-users (fathers) and researchers.			Abstract<br>Background<br>Methods<br>Results<br>Discussion
Mooses et al., 2021[4]<br>Estonia	To describe the design of a school-based PA intervention (Schools in Motion – SiM) to inspire and offer tips for designing and implementing PA-enhancing interventions within school systems.<br>Co-design and practice-to-evidence approach (through network building).	Not defined specifically. However, schools are considered as partners and experts whose feedback is thoroughly considered.<br>Networking and exchange of practices (both good practices and failures) were encouraged and supported throughout the pilot program through different school visits and seminars for school teams.	School teams (maximum 5 members, including school principal), school students, researchers, experts from sport and health sciences, education, social sciences, psychology and communication.	Not formally assessed, but a flexible and comprehensive program was developed which was well received by both schools and overall society.	*Co-create, co-design*<br>Abstract<br>Methods<br>Discussion
Ochieng et al., 2021[5]<br>UK (England)	To highlight partnership work with Black African migrant participants to co-create culturally sensitive resources and frameworks that will promote healthy weight maintenance in early childhood.<br>Partnership method through focus groups and workshops.	Co-production defined as an approach or strategy for developing person-centred services that meet the needs of individuals and communities. Identify a shift in thinking from needs and interventions defined and developed by service providers to a more participative process between experts and communities to generate and build meaning. Aims to achieve behavioural change through engaging end users.	Black African parents, health visitors, nutritionist and researchers.	Effectiveness not assessed.	*Co-create*<br>Title<br>Abstract<br>Background<br>Methods<br>Results<br>Discussion
Taggart et al., 2021[6]<br>Review – authors from UK	To explore lifestyle/obesity programs for adults with intellectual disabilities and to develop a draft logic model to implement and sustain lifestyle/obesity behaviour change in community settings for people with intellectual disabilities.<br>Co-production workshops.	Co-production not defined.	End users (adults with intellectual disabilities), senior management within specific organisations.	Effectiveness not assessed.	*Co-produce*<br>Abstract<br>Background<br>Discussion

Reference / Country	Aim / Methodology	What is meant by co-production in the development and evaluation of chronic disease prevention interventions?	Who is involved in the co-production of the development and evaluation of chronic disease prevention interventions?	How effective (process and impact measures) are chronic disease prevention programs developed using co-production processes?	Where in the paper is co-production (or other 'co-word') mentioned?
Ahmed et al., 2020[7]<br><br>US	Evaluation of 6-week co-designed fresh fruit and vegetable dietary intervention (Eat Fresh) targeted at low-income participants to improve dietary quality and perceptions of well-being.<br><br>Pilot study with participants enrolled in the Food Distribution Program on on the Flathead Reservation (Montana).	Not defined per se, but the process of co design is the Delphi method to gather the opinions and priorities of local food and nutrition stakeholders in order to co-design and implement an intervention that is place-based and culturally appropriate for the specific tribal context of the Flathead Reservation. The Delphi method proceeded with a series of focus group interviews to identify the priority populations, the priority health concerns, culturally relevant intervention methods, the intervention setting and duration, measurement of outcomes, and data analysis.	Community Advisory Board of local food and nutrition stakeholders (n=15) who live and work on the Flathead Reservation including Tribal elders, educators, enterprise representatives, clinical practitioners, policymakers, member of the Tribal Council.<br><br>Study team with previous research experiences in the community.	Paper reports on pre post intervention (on food procurement practices and a range of health-related outcomes).<br><br>Participants reported increased daily consumption of fruit and vegetables and improved dietary quality.<br><br>Intended and unintended consequences of a dietary intervention provide lessons in co-designing community-based programs	*Co-design*<br><br>Abstract<br><br>Key words<br><br>Background<br><br>Methods<br><br>Discussion
Beckerman Hsu et al., 2020[8]<br><br>US	To outline the process evaluation design for a 10-week family-centred intervention to prevent childhood obesity and promote family well-being.<br><br>Communities for Healthy Living was co-designed with low-income parents using Community Based Participatory Research (CBPR).	CBPR uses process evaluation methods to:<br><br>(a) Prioritize community members' power to adapt the program to local needs over strict adherence to intervention protocols.<br>(b) Share process evaluation data with implementers to maximize benefit to participants.<br>Ensure partner organizations are not overburdened.	Head Start staff members, low-income parents.	Protocol paper – effectiveness not assessed.	*Co-design*<br><br>Abstract
Bogomolova et al., 2020[9]<br><br>US	To evaluate the co-creation of a program to improve the healthfulness of food choices in supermarkets among consumers and to promote their well-being (A Healthy Choice).<br><br>Program underpinned by design thinking (DT) which uses co-design as a method.	Co-design defined as a scientific method of qualitative data collection aiming to include and empower stakeholder to contribute to the design process as experts of their unique experiences.<br><br>Five co-design workshops with consumers and staff (n = 32) to develop a consumer-centred program which included feedback on previous programs and creation of new program ideas incorporated into the development of the program.	Consumers and staff.	The program was effective in increasing consumer knowledge of healthier food choices (measured via public survey). Sales analysis showed mixed results: sales increased for promoted products in some categories, but there was no effect in others.	*Co-design, co-create*<br><br>Title<br><br>Abstract<br><br>Key Words<br><br>Background<br><br>Methods<br><br>Results<br><br>Discussion

Reference / Country	Aim / Methodology	What is meant by co-production in the development and evaluation of chronic disease prevention interventions?	Who is involved in the co-production of the development and evaluation of chronic disease prevention interventions?	How effective (process and impact measures) are chronic disease prevention programs developed using co-production processes?	Where in the paper is co-production (or other 'co-word') mentioned?
Brimblecombe et al., 2020[10]<br><br>Australia	To assess the effect of restricted unhealthy food promotion in remote Australian stores, specifically those items contributing most to free sugar sales, on food and beverage sales<br><br>Healthy Stores 2020 was a co-designed strategy to restrict merchandising of unhealthy food, (versus a control group of usual retail practice).	Not defined, but the strategy was co-designed with industry (with strategies to reduce merchandising of discretionary products on customer purchasing and business performance). Community organisation and expert working group provided input into what would or would not be considered acceptable and feasible to the community. These strategy components were considered further and refined over meetings between the ALPA operations manager and members of the research team.	Expert working group: industry/retailers (Arnhem Land Program Aboriginal Corporation, ALPA), ALPA nutritionist, study investigators, a government store licensing program person, Northern Territory government health and nutrition persons and a business and marketing academic.	Evaluation found that restricted merchandising of unhealthy foods and beverages allowed for complementary merchandising of healthier foods and beverages in a real-world store setting. This co-designed intervention can achieve both public health and business relevant gains.<br><br>Outcomes of interest were based on difference in weekly sales with the strategy compared with no strategy in:<br><br>a) Free sugar from all foods and beverages.<br>b) Targeted food or beverages.<br>Gross profit (AU$) using mixed models.	*Co-design*<br><br>Abstract<br><br>Background<br><br>Methods<br><br>Discussion
Castro et al., 2020[11]<br><br>Brazil	To identify requirements for designing a mobile app to encourage physical activity in a low-income community population of older people in Brazil.<br><br>Co design though qualitative focus groups.	Co-design defined as a technique where end users work in partnership with other stakeholders and researchers at all stages of intervention development (needs assessment, content definition, prototyping, testing and dissemination).<br><br>The processes included:<br><br>a) Baseline assessments.<br>b) Focus group with physically active older people and one with physically inactive older people<br>Design activities with both groups.	Community-dwelling adults (40-90 years), researchers	Reports on the results of the co design process:<br><br>Developing physical activity apps for older people should consider the following features: free application, simple interface, motivational messages using audio and visual information, sharing information among users, multimedia input and sharing and user customisation	*Co-design, co-create*<br><br>Abstract<br><br>Background<br><br>Methods<br><br>Discussion
Champion et al., 2020[12]<br><br>Australia	To summarise the co-design and user testing of a school-based, web-based cartoon intervention developed to concurrently prevent 6 key lifestyle risk factors for chronic disease among secondary school students: alcohol use, smoking, poor diet, physical	Not defined, but the process involved:<br><br>a) Consultation with adolescents (web-based survey) to identify knowledge gaps, attitudes, barriers and facilitators in relation to the risk factors.	Collaboration with students, teachers, and researchers with expertise relevant to the 6 lifestyle risk factors.	This paper reports on the outcomes of the co design process - the co-design process resulted in a six-module, evidence-informed program that uses interactive cartoon storylines and web-based delivery to engage students	*Co-design*<br><br>Title<br><br>Abstract<br><br>Background<br><br>Methods

Reference / Country	Aim / Methodology	What is meant by co-production in the development and evaluation of chronic disease prevention interventions?	Who is involved in the co-production of the development and evaluation of chronic disease prevention interventions?	How effective (process and impact measures) are chronic disease prevention programs developed using co-production processes?	Where in the paper is co-production (or other 'co-word') mentioned?
	inactivity, sedentary recreational screen time, and poor sleep.<br>Iterative co-design process.	b) Consultation with youth and experts on content and web development.<br>User testing of program with students and teachers to evaluate acceptability, relevance, and appeal to the target audience.			Discussion
Corr and Murtagh 2020[13]<br>Ireland	To assess the feasibility of involving girls in the co-creation of an activity program.<br>Mixed methods including questionnaires and focus groups to inform intervention design.	Co-creation not defined. Co-creation involved inclusion of students in the intervention design, providing insights into participants' capability, opportunity and motivation for change, using the Behaviour Change Wheel.	Students and researchers.	Involving students in co creation was feasible. Both intervention content and study procedures were feasible as demonstrated by successful recruitment, retention, adherence and acceptability.	*Co-create*<br>Title<br>Abstract<br>Background<br>Discussion
D'Addario et al., 2020[14]<br>Italy	To investigate the features important for engagement with a physical activity mobile app and the reasons for their importance.<br>Qualitative focus group methodology, with elements of co-design.	Co-design defined as the creative collaboration between researchers and end users, referring to their involvement in the design development process, as 'experts of their experience'.<br>A co-design pack was created according to a preliminary revision of the literature.	Target audience (people reporting sedentary lifestyles and willingness to improve PA) and researchers.	Paper reports on the themes that target audience believe are important to mobile apps.	*Co-design*<br>Abstract<br>Key words<br>Background<br>Methods<br>Discussion
Daly-Smith et al., 2020[15]<br>UK (England)	Description of the design of the Creating Active Schools Framework – and the process of multi stakeholder input .it describes a process of involving the whole of school in developing approaches to improve children's PA.<br>The aim of the current study was to co-develop a whole-school physical activity framework with multiple stakeholders, using the double diamond design approach (DDDA).	Experience based co-design methodology was used (DDDA). With DDDA, stakeholders progress through a four-stage reflective process to discover, define, develop, and deliver an innovative solution to a problem. The strength of this design approach is in the collaboration between multiple stakeholders within an innovative development process to produce an understanding greater than the sum of the individual parts.	Stakeholder groups - school leaders, teachers and other school staff, children/young people, parents/guardians, and wider stakeholders (e.g. active school coordinators, public health specialists).	Paper reports on process of developing the Creating Active Schools Framework, which was co designed from inception.	*Co-produce, co-design, co-develop*<br>Title<br>Abstract<br>Key words<br>Background<br>Methods<br>Discussion
De Rosis et al., 2020[16]<br>Italy	To investigate whether and how applying a co-production approach to an intervention for promoting healthy lifestyle among adolescents	A key aspect of co-production considers the roles of professionals and end users in the co-production process (in an equal and reciprocal	End users, researchers, intervention developers.	The co-production model was successfully implemented and appears to be effective. >5000 adolescents reached by 49 co-	*Co-produce, co-develop, co-design*<br>Title

Reference / Country	Aim / Methodology	What is meant by co-production in the development and evaluation of chronic disease prevention interventions?	Who is involved in the co-production of the development and evaluation of chronic disease prevention interventions?	How effective (process and impact measures) are chronic disease prevention programs developed using co-production processes?	Where in the paper is co-production (or other 'co-word') mentioned?
	could be effective and efficient (beFood).<br>Mixed methods using a single in-depth case study design involving field observation sessions and questionnaires.	relationship). Co-production acknowledges end users as potential partners.<br>Co-design is about improving the performance of existing public services by actively involving the service user in their design, evaluation and improvement.		producer adolescents, who reported behavioural changes (eating better and practicing more physical activity). Cost analysis showed that the co-production approach was efficient, producing relevant savings and potentially making available >3000 hours of professionals' time.	Abstract<br>Key words<br>Background<br>Methods<br>Results<br>Discussion
Gallegos et al., 2020[17]<br>Australia	This study evaluates the effectiveness of the Living Well Multicultural-Lifestyle Management Program, a culturally tailored programme to promote personal behaviours that ameliorate risk factors for and facilitate management of chronic disease in ethnic communities in Queensland, Australia.	No definition of co design was provided – it was detailed that the program was co designed and described in the following: Living Well Multicultural-Lifestyle Management Program was developed in consultation with the respective communities and multicultural health workers based on the specific needs of each community.	With target communities and Program staff.	Evaluation focused on delivery of program and lifestyle related measures (fruit and vegetable consumption, other food related behaviours, physical activity levels, weight, BMI, WC, WTHR and Blood pressure).<br>Conclusion - Engaging targeted communities in designing interventions focussed on healthy personal behaviours helps with delivery and implementation. Behavioural interventions should be culturally tailored to increase their effectiveness.	*Co- design*<br>Abstract
Hardt et al., 2020[18]<br>Australia	To describe the methodology, results and impact of a co-designed community-based, childhood overweight/obesity prevention program tailored to Māori & Pacific Islander cultures (Healthier Together).<br>Iterative, participatory and experience-based process guided by guidelines for Māori ethics to promote respect and equity.	Co-design in healthcare described as involving all relevant stakeholders, especially the target population, in developing an innovation. This empowers the target population by driving shared and equal decision-making and promoting ownership and satisfaction with the innovation. May be participatory, with target population directly engaged at multiple timepoints, and experience-based where it is driven by the lived experience of the users.	Co-design team: Māori & Pacific Islander health consumers, cultural advisors, health professionals and multidisciplinary researchers.	Authors report the process of co-designing the Healthier Together intervention.<br>Effectiveness not assessed.	*Co-design*<br>Title<br>Abstract<br>Key words<br>Background<br>Methods<br>Results<br>Discussion
Hidding et al., 2020[19]	To co-create a 24-hour movement behaviour tool for primary school children by:	No specific definition of co creation used. Methodology included the key	9-12 year olds and researchers.	Effectiveness not assessed.	*Co- create*<br>Title

Reference / Country	Aim / Methodology	What is meant by co-production in the development and evaluation of chronic disease prevention interventions?	Who is involved in the co-production of the development and evaluation of chronic disease prevention interventions?	How effective (process and impact measures) are chronic disease prevention programs developed using co-production processes?	Where in the paper is co-production (or other 'co-word') mentioned?
The Netherlands	1) examining children's perception of physical activity using the concept mapping method;<br><br>2) examining children's physical activity behaviour and the context of their behaviour using the photo voice method;<br><br>3) screening the literature on relevant questionnaire items regarding sleep and sedentary behaviour, and<br><br>4) assessing the content validity of the newly developed MyDailyMoves, together with children and researchers in the fields of child public health, measurement tool development, physical activity, sedentary behaviour and sleep.	populations' perception of physical activity using qualitative methods.<br><br>Concept mapping is a method in which group perceptions are examined using a qualitative data collection and a quantitative data analysis.<br><br>Children's physical activities, and their locations were examined using photovoice<br><br>A measurement tool (MyDailyMoves) was developed and existing questionnaires regarding sleep and sedentary behaviour were screened to include relevant items regarding these behaviours in the tool.			Abstract<br><br>Background<br><br>Methods<br><br>Discussion
Lems et al., 2020[20]<br><br>The Netherlands	To better understand the complexity of addressing health behaviour of adolescent girls with low SES (in developing their own health promotion materials).<br><br>Participatory action research approach.	Weekly small group sessions at school or a welfare organisation over 3 months provided opportunities for girls to share their stories and reflect on them. Creative methods were used (drawing, photo collages, making health promotion materials, cooking).	End-users (adolescent girls 12-15 years, with low SES) and policymakers of the Amsterdam Healthy Weight Program.	Comment on the importance of contextualised knowledge for tailored health promotion.<br><br>Effectiveness not assessed.	*Co-create*<br><br>Keywords<br><br>Methods<br><br>Results<br><br>Discussion
Martin et al., 2020[21]<br><br>Spain, Italy and UK	To describe the process of and findings from co-designing and prototyping components of the PEGASO Fit for Future (F4F) mHealth intervention for adolescents.<br><br>Qualitative methods (workshop, focus groups, questionnaires).	Co-design of an mHealth intervention for adolescents.<br><br>Adolescents attended a single workshop session to review early-version technology prototype, followed by a week-long testing of apps at home or school. Further developed prototypes tested for 2 weeks by participants. Feedback and development ideas collected.	End users: adolescents from different cultural backgrounds.	Feasibility of developing a complex mobile phone-based technological system applying principles of co-design to mHealth technology was feasible.	*Co-design*<br><br>Abstract<br><br>Keywords<br><br>Background<br><br>Methods<br><br>Results<br><br>Discussion

Reference / Country	Aim / Methodology	What is meant by co-production in the development and evaluation of chronic disease prevention interventions?	Who is involved in the co-production of the development and evaluation of chronic disease prevention interventions?	How effective (process and impact measures) are chronic disease prevention programs developed using co-production processes?	Where in the paper is co-production (or other 'co-word') mentioned?
Anselma et al., 2019[22]<br>Anselma et al., 2020[23]<br>The Netherlands	To describe how Youth-led Participatory Action Research (YPAR) and Intervention Mapping (IM), were combined in the Kids in Action study (which aims to improve health behaviours of 9–12-year old children living in a low socioeconomic neighbourhood in Amsterdam, by co-designing interventions with these children.<br>Participatory action research and intervention mapping.	Co-design is seen as participatory action research and intervention mapping	Youth involved in research process as co-researchers: from designing research question to intervention development, implementation and evaluation.<br>Collaborators in this study:<br>Child researchers<br>Academic researchers<br>Youth Policy managers	Effectiveness not assessed.	*Co design, co-create*<br>Title<br>Abstract<br>Results<br>Discussion
Nahar et al., 2020[24]<br>UK (England)	To enhance stakeholder' engagement, to implement lifestyle interventions for cardiovascular primary prevention, in disadvantaged populations and motivate uptake of NHS health checks.<br>Mixed methods to model co-production and implementation research.	Co-produce and co-design.<br>Iterative co-design with community representatives (community engagement).<br>Stage 1: explore the implementation context and co-produce the intervention (e.g. map stakeholders, map context, action planning by participants).<br>Stage 2: intervention rollout, recruitment and evaluation (stepped wedge, cluster RCT).	Stakeholder mapping to identify potential stakeholders and community representatives.	Protocol paper – effectiveness not assessed.	*Co-produce, co-design*<br>Abstract<br>Background<br>Methods<br>Discussion
Parder 2020[25]<br>Estonia	To pilot methods for the involvement and empowerment of adolescents in alcohol abuse prevention.<br>Participatory action research to capture and facilitate narratives among young people (13-15 years), where participants engaged in storytelling and co-creation of stories in digital form.	Co-creation not defined.	End-users, researcher.	Effectiveness not assessed.	*Co-create, co-produce*<br>Title<br>Abstract<br>Results<br>Discussion
Peiris-John et al., 2020[26]<br>New Zealand	To describe the process of engaging with young people and health practitioners, including digital service providers, to develop an	Co-design described as an adolescent-practitioner-researcher collaboration and partnership.	End-users: adolescents and young people, digital and health service	Effectiveness not assessed.<br>Student's perceived integrating access to digital health	*Co-design, co-create*<br>Title

Reference / Country	Aim / Methodology	What is meant by co-production in the development and evaluation of chronic disease prevention interventions?	Who is involved in the co-production of the development and evaluation of chronic disease prevention interventions?	How effective (process and impact measures) are chronic disease prevention programs developed using co-production processes?	Where in the paper is co-production (or other 'co-word') mentioned?
	intervention-integrated survey for adolescents.<br>Iterative qualitative research process: participatory co-design sessions.		providers, community stakeholders and researchers.	interventions into a large-scale youth health survey as acceptable and highly beneficial.	Abstract<br>Key words<br>Background<br>Methods<br>Discussion
Ruan et al., 2020[27]<br>Review – authors from Australia	To review the content, readability and interactivity of Australian websites on health behaviours and risk factors for NCDs relevant to adolescents.<br>Content analysis.	Not specified.	Not specified.	Discussion states: Understanding adolescents' preferences can be achieved through the process of co-design, to enable health organisations to engage with and understand individual needs of keys stakeholders.	*Co-design*<br>Abstract<br>Discussion
Skerletopoulos et al., 2020[28]<br>Greece	To describe the "Trikala Quits Smoking" initiative to enforce smoking legislation, creating a new social norm to counter national indifference to enforcing the law regarding smoking indoors.<br>Citizen co-creation and an 'all streams' inclusion approach.	Citizen co-creation described as adding accountability to behaviour change, whereby co-creators model new behaviour in the community, and reduce barriers to change for the rest of the community.	Local community and commercial stakeholders.	Social norms were positively impacted and the number of business complying with an indoor smoking ban increased. Citizen co-creation can have a role in the success and sustainability of a behaviour change program	*Co-create*<br>Title<br>Abstract<br>Key words<br>Methods<br>Discussion
Vallentin-Holbech et al., 2020[29]<br>Denmark	To investigate how young people perceived their participation in a co-creation process that involved multiple stakeholders developing a gamified virtual reality (VR) simulation targeted at adolescent users.<br>Participatory process guided by the Living Lab method: co-creation takes place in real-life settings, allowing for obtaining feedback and insights while experimenting.	Co-creation processes are guided by a systems perspective, which recognises the interrelationships between different parts of a system rather than focusing on any one part. Co-creation addresses the quality of stakeholder relationships by considering power sharing or participatory co-creation and using conflict as a positive force to overcome power structures.	End users (young people), developers of VR products, researchers.	Effectiveness not assessed.	*Co-create*<br>Title<br>Abstract<br>Key words<br>Methods<br>Results<br>Discussion
Corr and Murtagh 2019[30]<br>Ireland	To assess the feasibility of involving girls (15-17 years old) in the co-creation of an activity programme.	The Behaviour Change Wheel guided intervention design, providing insights into participants' motivations,	Target audience (teenage girls) and researchers.	This paper reports on the results of the delivery of the co-designed PA program, in terms of attendance, retention and effectiveness	*Co-create*<br>Abstract<br>Background

Reference / Country	Aim / Methodology	What is meant by co-production in the development and evaluation of chronic disease prevention interventions?	Who is involved in the co-production of the development and evaluation of chronic disease prevention interventions?	How effective (process and impact measures) are chronic disease prevention programs developed using co-production processes?	Where in the paper is co-production (or other 'co-word') mentioned?
	Focus groups and questionnaire was used (formative research) for intervention design and assessment of intervention practicability.	capabilities and opportunities for change.		(resulting in an increase of average daily steps).	Discussion<br>Conclusion
Fehring et al., 2019[31]<br>Australia	To evaluate a co-designed multi-strategy health promotion initiative, implemented over 12 months from 2017 to 2018.<br>Mixed methods.	Not specifically mentioned – just that all components of the project were co designed through Community Advisory Committees.	Project staff, Aboriginal shire councils, community leaders, organisations and community members	Effectiveness was measured by:<br>Community readiness to address sugary drink consumption increased in 2/3 communities.<br>High awareness of social marketing campaign messaging (56‑94%).<br>Increased availability of drinking water in all communities. Water sales as a proportion of total drink volume sales increased by 3.1% ($p<0.001$) while sugary drink volume sales decreased by 3.4% ($p<0.001$).<br>No comparison group to determine impact of co-design	*Co-design*<br>Abstract<br>Methods<br>Discussion
Folkvord 2019 [32]	Protocol paper for a project testing the effects of promotion techniques on children's fruit and vegetable intake	Co-creation – through focus groups (but no further information)	Children and Parents	The basis of the paper is that using co-creation has been proven to be a very effective methodology to create innovations, by increasing external and ecological validity.	*Co-create*<br>Methods
Fournier et al., 2019[33]<br>The Netherlands	To describe the approach used to develop the Walking with Poles program (WWPP) and outline the contents of the program. It also includes the implementation outcomes of this program among seven community organizations for older adults in the context of a program evaluation.	Intersectoral co-construction or collaboration is an increasingly popular approach in the development of socially innovative programs.<br>The development of the WWPP is based on a six-phase co-constructive approach to develop health promotion programs and tools for older adults:<br>(1) definition; (2) conceptualization; (3) creation and validation; (4) pilot test; (5) evaluation and generalization; and (6) consolidation.	Steering Committee (health promotion planner, researcher and masters student) and stakeholders (older adults / target audience).	To date, the five stages of this co-construction project have been successfully operationalized through the WWPP. The program was implemented by seven community organizations. It reached 76 older adults whose characteristics are typical of this type of community-based group physical activity program	*Co-construct*<br>Abstract<br>Background<br>Discussion

Reference / Country	Aim / Methodology	What is meant by co-production in the development and evaluation of chronic disease prevention interventions?	Who is involved in the co-production of the development and evaluation of chronic disease prevention interventions?	How effective (process and impact measures) are chronic disease prevention programs developed using co-production processes?	Where in the paper is co-production (or other 'co-word') mentioned?
Gillespie et al., 2019[34]<br>UK (Scotland)	Study protocol of a feasibility study to translate the Healthy Habits, Happy Homes (4H) a home based, preschool childhood obesity prevention intervention to families with preschool children living in a Scottish community experiencing health, social and economic inequalities (4H Scotland).<br>Mixed methods using community based participatory research (CBPR) and co-production approaches.	Elements of co-production and community based participatory research used to adapt the original 4H study to maximise 4H's cultural relevance for target population.<br>Participatory approaches involve potential participants in study processes and provide insight to the context in which the research outputs will be applied. Co-production can help to ensure that the people themselves, are empowered and enabled to be involved.<br>Features of both co-production and CBPR were applied to engage and involve key stakeholders in the research process at a local level.<br>Co-production is underpinned by key values of equal and reciprocal relationships, being assets based and 'doing with, not to'.	Target audience and planners.	Process evaluation will be used to offer detail in relation to the participatory, co-production approach.	*Co-produce*<br>Abstract<br>Background<br>Methods<br>Results<br>Discussion
Gillespie et al., 2019[35]<br>UK (Scotland)	To describe the process of combining Analysis Grid for Environments Linked to Obesity (ANGELO) with community engagement, qualitative and co-production methods to promote local strategies around child healthy weight (CHW) and to highlight steps to engage local people in developing a community CHW action plan around two school communities in Dundee, Scotland.<br>Co-production and qualitative methodology combined with ANGELO: 1) Mapping and engaging, 2) Analysing and prioritising, 3) Shaping a community healthy weight action plan.	No definition of co –production is provided – but focus group discussions were used.<br>The Analysis Grid for Environments Linked to Obesity (ANGELO) model provides a systematic method for engaging with a community to help them to identify their priorities for healthy weight in their community leading to an action plan which gives a route to making necessary changes and informing policy decisions.	Target audience participants.	Effectiveness not assessed.	*Co-produce*<br>Title<br>Abstract<br>Background<br>Methods<br>Discussion

Reference / Country	Aim / Methodology	What is meant by co-production in the development and evaluation of chronic disease prevention interventions?	Who is involved in the co-production of the development and evaluation of chronic disease prevention interventions?	How effective (process and impact measures) are chronic disease prevention programs developed using co-production processes?	Where in the paper is co-production (or other 'co-word') mentioned?
Goffe et al., 2019[36]<br>UK (England)	To explore the feasibility of working with a wholesale supplier to co-design and deliver, and to assess the acceptability of, an intervention to promote smaller portions in Fish & Chip shops.<br>Before-and-after study.	Co design intervention is described as a partnership that included determining roles and responsibilities etc.	Wholesale supplier of fish and chips and researcher.	Evaluation found it feasible to co-design and deliver an intervention to promote smaller portions with a commercial partner and the intervention was acceptable to both Fish & Chip shops and their customers.	*Co-design*<br>Abstract<br>Background<br>Methods<br>Discussion
Gould et al., 2019[37]<br>Australia	To explore the feasibility and acceptability of a) a co-designed multi-component intervention for health providers at Aboriginal Medical Services (AMSs) in culturally-targeted pregnancy specific smoking cessation care and b) the study design.<br>Randomised stepped wedge cluster design.	The intervention was designed collaboratively after in depth community consultation, under the guidance of a Stakeholder and Consumer Aboriginal Advisory Panel to produce a suite of training and educational resources for HPs and the pregnant Indigenous women they consult. Based on the Behaviour Change Wheel (BCW) and Theoretical Domains Framework (TDF).	Health Providers and researchers.	Evaluation of the intervention: Feasibility was assessed through recruitment and retention rates of both pregnant women (12-weeks) and health professionals (end of study) as well as the potential to improve women's quit rates. Qualitative interviews with staff post-trial explored acceptability of the intervention and study, based on capability, opportunity and motivation from the BCW.	*Co-design*<br>Abstract
Hoeeg et al., 2019[38]<br>Denmark	To analyse how the design-based research (DBR) process of co-design was shaped by the meeting between the fields of health promotion research, municipal professionals, and families and how it affected the social effectiveness of the intervention development.<br>Design based research.	DBR is an innovative methodology for co-creation - it typically consists of a needs assessment and an ideation phase, followed by feasibility and pilot tests, before implementing a designed intervention. It is characterized by repeated loops of designing, enactment, analysis, and redesign.	Co-creation partnership between the research centre and a municipality. In the partnership, DBR was chosen as the methodology to generate co-design collaboration between local professionals, families, and researchers.	Descriptive evaluation of the co creation process and perceptions by stakeholders.	*Co-design; co-create*<br>Title<br>Abstract<br>Key words<br>Background<br>Methods<br>Results<br>Discussion
Lems et al., 2019[39]<br>The Netherlands	To understand perceptions of health and health-promotion strategies among adolescent boys from disadvantaged neighbourhoods to identify tailored health promotion opportunities (The Healthy Lifestyle Project).	Co-creation using participant observations, and 8 co-creation sessions with sport and cooking activities over a 3-month period, aiming to challenge boys to reflect on their own lifestyles and advise policymakers and other professionals about health promotion that fit their daily realities.	Hard-to-reach population (adolescent boys from disadvantaged neighbourhoods) and policymakers of the Amsterdam Healthy Weight Program.	Effectiveness not assessed.	*Co-create*<br>Abstract<br>Methods

Reference / Country	Aim / Methodology	What is meant by co-production in the development and evaluation of chronic disease prevention interventions?	Who is involved in the co-production of the development and evaluation of chronic disease prevention interventions?	How effective (process and impact measures) are chronic disease prevention programs developed using co-production processes?	Where in the paper is co-production (or other 'co-word') mentioned?
	8 co-creation sessions held using a qualitative, participatory research approach.				
Mammen et al., 2019[40]<br>USA	To provide a greater understanding about health messaging directed at rural, low-income families.<br>Uses participatory action research.	Co-creation of health messages, through focus groups and interviews with mothers, and telephone interviews with stakeholders.	Rural, low-income mothers and community stakeholders.	Feasibility of developing co-created health messages demonstrated.	*Co-create*<br>Abstract<br>Background<br>Methods<br>Results<br>Discussion
Mistura et al., 2019[41]<br>Canada	To examine the impact of a contextually feasible evidence-informed nudge intervention on food purchasing behaviour of older adolescents in a university residence cafeteria.<br>Qualitative research: surveys and focus groups.	Co-design: Formative research was conducted that involved surveying students and conducting focus groups with food services staff to help inform which nudges to trial in this real-life setting with this target audience.	End-users: students and service staff informed the intervention content. Intervention co-designed with service staff and researchers.	The impact of this intervention on older adolescent vegetable purchasing remains in question but the effect sizes were promising	*Co-design*<br>Abstract<br>Methods<br>Discussion
Morgan et al., 2019[42]<br>UK (Wales)	To gather views from preadolescent girls, parents, teachers and stakeholders in order to co-produce a multicomponent school-based, community linked PA intervention programme.<br>Qualitative research: focus groups, in-depth interviews and stakeholder engagement events.	Co-production: involving the target audience in the design and implementation of an intervention.<br>School students and parents participated in (separate) focus groups, the head teacher and one other teacher participated in a face-to-face interview, and stakeholders participated in face-to-face meetings (school, local authority, policy and national governing representatives).	School students (target audience), parents, teachers and policy-representatives.	Data show numerous barriers to intervention uptake and continued PA participation when designing a school-based, community-linked intervention. Adopting a co-production approach, this formative work highlights potential strategies for overcoming these barriers.<br>Findings directed development and implementation of the CHARMING role model intervention and informed creation of an intervention logic model.	*Co-produce*<br>Abstract<br>Background<br>Methods<br>Discussion
Ojo et al., 2019[43]<br>UK (England)	To develop a tailored intervention to break up and reduce workplace sitting in desk-based workers.	Semi-structured interviews with 25 office workers. Qualitative analysis using COM-B model of behaviour to identify 39 behaviour change techniques as potential active	Researchers and end-users (i.e. office workers).	Feasibility, but not effectiveness assessed.	*Co-create*<br>Abstract<br>Method

Reference / Country	Aim / Methodology	What is meant by co-production in the development and evaluation of chronic disease prevention interventions?	Who is involved in the co-production of the development and evaluation of chronic disease prevention interventions?	How effective (process and impact measures) are chronic disease prevention programs developed using co-production processes?	Where in the paper is co-production (or other 'co-word') mentioned?
	Qualitative research: semi-structured interviews.	components for an intervention to break up sitting time in the workplace.			
Partridge et al., 2019[44]<br>Australia	To co-design a bank of text messages that are evidence-based, acceptable, and engaging for adolescents.<br>Iterative mixed methods process.	Workshop with researchers and health professionals experienced in working with adolescents.<br>Review of initial bank of text messages by adolescent research assistant.<br>User acceptance testing with 13-18-year old adolescents.	Adolescents, health professionals and researchers, including a 19-year old research assistant.	Effectiveness not assessed but will be in a RCT. Authors state that this study may guide other researchers or health professionals seeking to engage adolescents in the co-design of health promotion or intervention content.	*Co-design*<br>Title<br>Abstract<br>Keywords<br>Background<br>Methods<br>Discussion
Rutten et al., 2019[45]<br>Review – using a German case study, authors from Germany, Switzerland, Australia, The Netherlands, USA and Canada	To introduce a system model that describes and explains co-production of active lifestyle.<br>Literature review and commentary.	A system model explains the relational actions between four key actors that shape interactive social practices for the co-production of active lifestyles.	Four key actors: population groups, professionals, policymakers and researchers.	Effectiveness not assessed.	*Co-produce*<br>Title<br>Abstract<br>Keywords<br>Background<br>Methods<br>Results<br>Discussion
Santina et al., 2019[46]<br>Lebanon	To describe the development of the IMove30+ programme and outline lessons derived from the authors' experience using intervention mapping.<br>Participatory framework.	In-depth involvement of stakeholders in all steps of planning, development and implementation.	Planning committee (school leaders, school staff members – nurse, supervisors, teachers) and researchers.	Effectiveness not assessed.	*Co-design, co-develop, co-implement*<br>Abstract<br>Discussion
Buckley et al., 2018[47]<br>UK (England)	To report process data from the participatory co-development phase of an exercise referral scheme.<br>Participatory research with multiple stakeholders.	Five development group meetings to facilitate the iterative development of the intervention; plus an online survey to confirm agreement of intervention components.	Public health commissioners, fitness centre area manager, general practitioner, health trainer coordinator, patients, academic experts.	The multidisciplinary co-development process highlighted cultural and pragmatic issues related to exercise referral provision, resulting in an evidence-based intervention framework designed to be implemented within existing infrastructures.	*Co-development; co-produce*<br>Title<br>Abstract<br>Background<br>Results

Reference / Country	Aim / Methodology	What is meant by co-production in the development and evaluation of chronic disease prevention interventions?	Who is involved in the co-production of the development and evaluation of chronic disease prevention interventions?	How effective (process and impact measures) are chronic disease prevention programs developed using co-production processes?	Where in the paper is co-production (or other 'co-word') mentioned?
					Discussion
Buckley et al., 2019[48]<br>UK (England)	To explore the preliminary effects and acceptability of a co-produced physical activity referral intervention.<br>Longitudinal pre-post design.	The co-produced intervention aimed to support participants to make gradual, sustainable changes to their PA levels.	The co-produced intervention was piloted in 1 of 11 fitness centres who staff were involved in the co-production phase (Buckley et al., 2018).	Significant improvements in participants' MVPA and cardiometabolic health profile were found following the co-produced PA intervention.	*Co-produce*<br>Title<br>Abstract<br>Keywords<br>Methods<br>Discussion
Guell et al., 2018[49]<br>UK (England)	To develop a typology of older people's motivations and lifelong habits of being active as a starting point to co designing active ageing strategies<br>Mixed methods study.	No description of what is meant by co design, but the methods used to gather information from the target audience was semi structured interviews	Target audience – older people	Effectiveness not assessed.	*Co design; co-develop*<br>Abstract<br>Results
Lombard et al., 2018[50]<br>Australia	To understand how young adults engage and utilise social media in relation to health and healthy eating.<br>Protocol.	An evidence-based approach drawing expertise and knowledge from several disciplines was used. As well as co-creation of the intervention content with the target group and expert stakeholders.	Industry partners, experts, young adults using social media at least twice daily,	Effectiveness not assessed – protocol paper.	*Co-create, co-design*<br>Abstract<br>Background<br>Methods<br>Discussion
McKay et al., 2018[51]<br>Canada	To evaluate the effectiveness of a PA intervention (Choose To Move) during the pilot and initial scale up phases on PA, mobility, and social connectedness among older adults in BC, Canada.<br>Type 2 hybrid effectiveness-implementation study design.	Co-design of the PA intervention for older adults resulted from a series of four, three hour in-person workshops with an 'older adult action committee' (15 government, NGO, not-for-profit, academic, and health authority stakeholders from across British Columbia). Researchers conducted a review of the literature to guide committee discussions based on evidence.	End-users (those who deliver an intervention and those who will receive the intervention). For this study, co-design included those who would deliver the intervention (BC Ministry of Health and community partners).	PA increased (baseline-3 months) in younger (60–74 yrs.; + 1.6 days/week; $p < 0.001$) and older (≥75 yrs.; + 1.0 days/week; $p < 0.001$) participants. Increase sustained at 6 months in younger participants only. Social exclusion indicators declined significantly in younger group. Mobility and strength improved significantly in both groups at 6 months.<br>Conclusion: a partner-based health promotion intervention can be effectively implemented across settings to enhance PA, mobility	*Co-design*<br>Title<br>Abstract<br>Background<br>Methods

Reference / Country	Aim / Methodology	What is meant by co-production in the development and evaluation of chronic disease prevention interventions?	Who is involved in the co-production of the development and evaluation of chronic disease prevention interventions?	How effective (process and impact measures) are chronic disease prevention programs developed using co-production processes?	Where in the paper is co-production (or other 'co-word') mentioned?
				and social connectedness in older adults.	
Partridge and Redfern, 2018[52]<br>Review – authors from Australia	To review evidence supporting effective engagement in digital interventions as a critical factor in the adoption of healthy dietary behaviours in adolescents within the current "digital world".<br>Narrative review.	Co-design or participatory design in public health is defined as the systematic co-creation, with those affected by the issues being studied, for the purpose of developing new strategies, programs, policies.	End-users (those affected by the issues being studied).	Effectiveness not assessed. Conclusion: incorporating strategies such as co-design with end-users may optimise adolescent engagement with digital health interventions.	*Co-design*<br>Abstract<br>Background<br>Discussion
Raeside et al., 2018[53]<br>Review – authors from Australia	To evaluate published eHealth behaviour change interventions targeting cardiovascular disease risk factors in adolescents, which utilise a co-creation process.<br>Narrative review.	Active process of engagement which creates awareness about CVD prevention and adolescents are directly involved in solution generation.	Adolescents, researchers, stakeholders.	Co-creation is one of the strategies that can increase engagement and effectiveness of cardiovascular disease prevention interventions in adolescents.	*Co-create*<br>Title<br>Keywords<br>Abstract<br>Background<br>Methods<br>Results<br>Discussion
Street et al., 2018[54]<br>Australia	To trial and evaluate a storyboard approach to engage Australian Aboriginal people in health policy priority setting.<br>Deliberative community forum.	Deliberative forum co-constructed by the research team and two Aboriginal community organisations and moderated by an independent Aboriginal facilitator. Storyboard provided a frame for discussion with participants.	Researchers, community stakeholders, Aboriginal participants recruited through widely accessed Aboriginal community Facebook page and word of mouth.	The group identified policies they believed governments should prioritise, including strategies to combat racism and provide local supports and outlets for young people.	*Co-construct, co-create, co-produce*<br>Abstract<br>Methods<br>Discussion
Taggart et al., 2018[55]<br>Review – authors from UK	To review recent developments in health promotion initiatives for chronic disease prevention in adults with intellectual and developmental disabilities, targeting type 2 diabetes.<br>Narrative review.	This review identified three programs adapted for this population. Each employed co-design and co-production techniques with stakeholders.	Adults with intellectual and developmental disabilities, their carers and 'other stakeholders'.	Health promotion and wellness initiatives need to be tailored and reasonable adjustments to address cognitive impairments and communication difficulties in this population.	*Co-design, co-develop; co-produce*<br>Abstract<br>Discussion
Te Morenga et al., 2018[56]	To describe the integration of codesign and Maori research	Primary objective was to enable Maori partners to lead the conceptualisation, design, implementation and interpretation of research outcomes,	University academics and a leading Māori health promotion organisation (Toi Tangata). The partnership was involved	Effectiveness not formally assessed. Researchers state some challenges faced and important	*Co-design*<br>Title

Reference / Country	Aim / Methodology	What is meant by co-production in the development and evaluation of chronic disease prevention interventions?	Who is involved in the co-production of the development and evaluation of chronic disease prevention interventions?	How effective (process and impact measures) are chronic disease prevention programs developed using co-production processes?	Where in the paper is co-production (or other 'co-word') mentioned?
New Zealand	approaches to deliver a mHealth lifestyle app for Maori people.<br><br>Qualitative. Participative co-design approach. Focus groups with participants for formative research and discussion with health promotion organisation to develop prototype mHealth app.	adapting a participatory co-design cycle to identify and understand the needs and desires of the community regarding health and well-being and their knowledge and ideas for solutions to meet those needs.	throughout all aspects of the project (developing research question, designing, evaluation, implementing and dissemination).<br><br>Maori participants were recruited for focus groups to assist with the design.	aspects critical to successful co-design, e.g.,<br><br>Tensions due to different priorities and expectations, e.g. timeframes set were not easily achievable.<br><br>Convincing funders of merits of co-design remains difficult. To secure funding research parameters, timelines and milestones are needed. These restrictions caused tension within the co-design process at times.<br><br>Building relationships and trust took time but was crucial for the project.	Abstract<br><br>Keyword<br><br>Method<br><br>Results<br><br>Discussion
Verbiest et al., 2018[57]<br><br>New Zealand	To determine the effect of a co-designed, culturally tailored lifestyle mHealth tool (app and website) on lifestyle behaviours (PA, nutrition, smoking and alcohol consumption).<br><br>RCT protocol paper.	Joint decisions were made about trial design, including the community-based cluster trial design, the control condition, primary and secondary outcome measures, recruitment methods, and timelines.<br><br>The mHealth tool was reported to be co-designed but this is reported elsewhere (Te Morenga et al., 2018).	M'ori and Pasifika community representatives and academics.	Effectiveness not assessed.	*Co-design*<br><br>Title<br><br>Abstract<br><br>Keywords<br><br>Method
Durl et al., 2017[58]<br><br>Australia	To explore co design as a method for actively involving young consumers in the design of an alcohol education program (that incorporates a virtual reality component).<br><br>Data collection included – written feedback, presentation of ideas, and facilitation and observation of young person ideas through a session/ workshop.	Six step framework for co-design, with active involvement of the target audience in content development of a new alcohol education program: 1. Resourcing, 2. Planning, 3. Recruitment, 4. Sensitising, 5. Facilitation, 6. Evaluation.<br><br>The creative and iterative approach typically included: 1. exploration of the user experience within their context; 2. ideation of design concepts relevant to the underlying project; 3. reflection on the concepts, including prototyping	Researcher and young person	This paper reports on the process of the six steps and found that they worked well in involving young people as co-designers with a number of modifications made to the six steps to make them more effective.<br><br>The effectiveness of the produced outcomes of the project have not been evaluated.	*Co-design*<br><br>Title<br><br>Abstract<br><br>Background<br><br>Methods<br><br>Results<br><br>Discussion

Reference / Country	Aim / Methodology	What is meant by co-production in the development and evaluation of chronic disease prevention interventions?	Who is involved in the co-production of the development and evaluation of chronic disease prevention interventions?	How effective (process and impact measures) are chronic disease prevention programs developed using co-production processes?	Where in the paper is co-production (or other 'co-word') mentioned?
		and testing; 4. implementation of the final design.<br><br>Co-design allows users to "become part of the design team as 'experts of their experiences'". Based on the tradition of participatory design, enables users to contribute their personal wants and needs during the design process.<br><br>Co-creation is generally considered to be any instance of creativity shared by multiple people during service design, while co-design is narrower, referring to the close collaboration between designers and end-users during the service design process. The aim of co-design is to empower non-designers, representing potential end-users, to incorporate their unique experiences into the design process and contribute innovative ideas.			
Hawkins et al., 2017[59]<br><br>UK (Wales and Scotland)	To present the framework for co-production and prototyping which was used to guide the adaptation of the ASSIST smoking prevention intervention to develop detailed content and delivery processes for two new peer-led drug prevention interventions, one as an adjunct to the ASSIST intervention (+Frank) and the other a standalone drug prevention intervention (Frank friends).<br><br>Descriptive study.	Three-stage framework extends current guidance on intervention development by providing step-by step instructions for co-producing and prototyping an intervention's content and delivery processes prior to piloting and formal evaluation.<br><br>1) Evidence review and stakeholder consultation;<br><br>2) Co-production;<br><br>3) Prototyping<br><br>In stage 2 - Co-production of intervention content took the form of an action research cycle over a series of meetings of the intervention development group in which findings from stage 1 were considered, ideas were presented by all members, feedback on ideas sought, refinements	Research team and key stakeholders	Effectiveness not assessed.	*Co-produce*<br><br>Title<br><br>Background<br><br>Methods<br><br>Results<br><br>Discussion

Reference / Country	Aim / Methodology	What is meant by co-production in the development and evaluation of chronic disease prevention interventions?	Who is involved in the co-production of the development and evaluation of chronic disease prevention interventions?	How effective (process and impact measures) are chronic disease prevention programs developed using co-production processes?	Where in the paper is co-production (or other 'co-word') mentioned?
		made and presented again, until final content was agreed. Five face-to-face meetings were held over the course of a four-month period. These were supplemented by communications via email where face-to-face meetings were not possible, or when matters arose that required discussion between meetings.			
Janols and Lindgren, 2017[60]<br>Sweden	To define and develop a methodology for co-designing theory-based behaviour change systems for health promotion that can be tailored to the individual.<br>Theories from two research fields were combined with a participatory action research methodology. Two case studies applying the methodology were also conducted. The methodology has strong emphasis on the target groups participation in the design process.	Checklist has been developed that can be used to explore with participants (which seems to then satisfy a process of co design if this information is collected from participants):<br>1.(diversity of) attitudes towards the targeted activity in focus and technology,<br>2. (diversity of) attitudes towards, and desires regarding the technology's potential pro-active behaviour such as encouraging and reminding messages,<br>3. (diversity of) attitudes, and desires regarding passive, summative communication of accomplishments,<br>4. (diversity of) attitudes, and desires regarding embedding social aspects and features, and<br>5. (diversity of) attitudes and desires regarding the system's potential agency and transparency.	End-users.	Effectiveness not assessed.	*Co-design*<br>Title<br>Background
Leask et al., 2017[61]<br>UK (Scotland)	To document and evaluate the co-creation of a public health intervention to reduce sedentary behaviour in older adults (Are you up for it?).<br>Used interactive co-creation workshops informed by Participatory and Appreciative	Co-production is not mentioned. Used 'co-creation' to develop a tailored intervention.<br>10 interactive workshops: using an iterative process (each workshop built on the discussion in the previous; research fieldwork tasks were conducted between workshops and	End-users (community-dwelling older adults) and academic researchers attended 10 interactive co-creation workshops together.	The co-creation approach was feasible, but effectiveness was not evaluated.<br>Future work should aim to develop principles and recommendations to ensure a scientific and reproducible co-creation process.	*Co-create*<br>Title<br>Keywords<br>Background<br>Methods

Reference / Country	Aim / Methodology	What is meant by co-production in the development and evaluation of chronic disease prevention interventions?	Who is involved in the co-production of the development and evaluation of chronic disease prevention interventions?	How effective (process and impact measures) are chronic disease prevention programs developed using co-production processes?	Where in the paper is co-production (or other 'co-word') mentioned?
	Action and Reflection methodology. Quantitative content analysis.	used for discussion in the following workshop, e.g. interviewing peers, gathering images, developing prototypes of intervention elements).			Discussion
Perignon et al., 2017[62]<br>France	To describe the co-construction and evaluation of a healthy eating intervention among participants in socioeconomically deprived situations.<br>A co-construction approach (integrating health promotion principles and social cognitive theory)	Workshops (5x2-hours) were held with 96 socio-economically disadvantaged individuals to develop the protocol and tools of the intervention and evaluation process. An iterative co-construction process was followed Semi-structured interviews and a group interview were conducted one month after the last workshop.	End-users (socio-economically disadvantaged individuals) recruited from community and health care centres.	Intervention and control groups compared. The workshops helped participants plan and rationalise their food purchases better. The nutritional quality of the experimental purchases increased but energy cost did not, showing that the co-constructed prevention program can favourably change food purchasing behaviours at no additional cost.	*Co-construct*<br>Title<br>Abstract<br>Background<br>Method<br>Results<br>Discussion
Van den Heerik et al.,2017[63]<br>The Netherlands	To explore the public's engagement with a tobacco social media campaign.<br>Corpus-linguistic approach to analysing messages.	Co-creation as a way to enable target audience members to become active campaign producers. Dutch Cancer Society developed a public health media campaign around tobacco. As part of the campaign the Cancer Societ developed the first part of the slogan "smoking is sooo…" and the public were able to contribute to the second part of slogan via social media. This contribution was dubbed as co-creation.	Researchers and public.	Co-creation provides the target audience with an opportunity to disseminate campaign messages from their own perspective, but at the same time a co-creation strategy risks diluting the intended campaign message.	*Co-create*<br>Title<br>Abstract<br>Background<br>Discussion
Verloigne et al, 2017[64]<br>Belgium	To develop, implement and evaluate physical activity-promoting interventions in co-creation with adolescent girls.<br>Mixed methods. The intervention was quasi-experimental with pre-post data collection. There were also focus groups with students in the co-creation group.	Co-creation of a PA intervention in schools. Groups were created with researchers and volunteer students. Researchers guided students to ensure the intervention was evidence-based, safe, realistic etc and assisted with the implementation of the intervention but decisions were jointly made with students. A different intervention was conducted at each school since the co-creation groups were different.	Researchers and adolescent girls (year 10) at vocational and technical education schools.	Pilot shows creating a co-creation group with adolescent girls is feasible. Girls said they enjoyed being involved in planning and would participate again.<br>There was a significant intervention effect on self-efficacy ($\beta = 0.91$; standard error (SE) = 0.23; $p < 0.001$) and extra-curricular sports participation ($\beta = 0.73$; SE = 0.09; $p < 0.001$).	*Co-create*<br>Title<br>Abstract<br>Background<br>Method<br>Results<br>Discussion
Yuan et al., 2017[65]	To analyse the ideation process of the co-creation process from	Co-creation was described as a collaborative process between people	17 partners from more than four different EU countries such as	Discussion focused on the functions/features that older adults	*Co-create*

Reference / Country	Aim / Methodology	What is meant by co-production in the development and evaluation of chronic disease prevention interventions?	Who is involved in the co-production of the development and evaluation of chronic disease prevention interventions?	How effective (process and impact measures) are chronic disease prevention programs developed using co-production processes?	Where in the paper is co-production (or other 'co-word') mentioned?
Four European Union countries	workshops with multiple stakeholders (researchers, technology providers, industry and care providers) to determine what extent this process supports technology adoption of older adults to promote PA.<br><br>Qualitative paper - Description of the collaborative process.	with shared goals but different expertise and skills.<br><br>The paper comments on three collaborative processes;<br>• End user value creation process:<br>• Stakeholder value creation process<br>Encounter process	knowledge providers (research institutes, universities), technology providers (sensors technologies, prediction software, intervention mechanisms), multiplicators (insurance companies, standardization organizations, etc. who are able to multiply the impact of the project in long term), and solution operators (clinics, rehabilitation centres and home care providers and older adults (users)).	would experience, not the motivation to purchase the product service system or how the technologies would be placed in the older adult's life.<br><br>Co-creation processes helped to determine what role the product will play and at a systemic level dealt with the interaction with a product and how to convert technology to an intended feature. The co-creation process did not discuss the process of ownership. As the project was in an early phase, no prototypes were made. A co-creation process needs to be applied when moving forward with the project.	Title<br>Abstract<br>Keywords<br>Background<br>Methods<br>Results<br>Discussion
Chau et al., 2016[66]<br><br>Australia	To describe formative research undertaken in regard to the perceptions of a Sit Less Move More Program in an Australian Emergency Call Centre. The Sit Less Move More Program was co designed with the management team of the call centre.<br><br>Mixed methods.	Unclear – it details that the program was co-designed with the management team of the centre but the process of the co design is not provided as the paper focuses on perceptions of the program by the employees	Researchers and the management team of the call centre (noting that it was not the employees who were the intended target)	Effectiveness not assessed.	*Co-design*<br>Abstract<br>Discussion
Eyles et al., 2016[67]<br><br>Review – authors from New Zealand	To describe the methods and processes used in the co-design of mHealth interventions.<br><br>Systematic review.	Co-design is a process in which targeted end users and other relevant stakeholders form a partnership with researchers and work together on all aspects of intervention development, from needs assessment to content development, pilot testing and dissemination.<br><br>The iterative nature of co-design fits well when collaborating with minority and indigenous populations because this approach allows for conceptual or tool re-developments and refining	One of the review questions was: Stakeholders—is it clear who was involved in the co-design, and do you know all that you need to about the participants?<br><br>The number of total individual participants involved in formative development ranged from approximately 10 to ~1000.<br><br>Type of participants and other stakeholders varied by study, but representatives from the target	Intervention effectiveness was not assessed by any of the studies in the review	*Co-design*<br>Title<br>Abstract<br>Key words<br>Background<br>Methods<br>Results<br>Discussion

Reference / Country	Aim / Methodology	What is meant by co-production in the development and evaluation of chronic disease prevention interventions?	Who is involved in the co-production of the development and evaluation of chronic disease prevention interventions?	How effective (process and impact measures) are chronic disease prevention programs developed using co-production processes?	Where in the paper is co-production (or other 'co-word') mentioned?
		based on the social cultural needs of partnership groups.<br>The co-design process is very similar to community-based participatory research (CBPR): 1. it is participatory, 2. there is cooperation between partners, 3. there is co-learning with mutual exchange of information between partners, 4. it involves systems development and sustainability and builds on the strengths of the community, 5. it is empowering due to shared decision-making across all aspects, 6. there is implementation of an intervention based on the findings, 7. there is recognition of the community as a social setting not just a physical one, 8. long-term commitment is required by all partners.<br>Six phases of design process: 1. opportunity identification, 2. generation of explicit and implicit knowledge, 3. identification of needs and desires, 4. description of delivery requirements, 5. envisaging the intervention and 6. prototype testing, pilot testing and evaluation.	population or clinical group for which the intervention was intended were always included (at a minimum).<br>Other stakeholders involved in intervention design (across all studies) were carers for those with clinical conditions; relevant clinical and/or public health practitioners; service providers; information technology experts (e.g., software programme developers and Web designers); behavioural experts; students; project managers; elders relevant to the culture of the intended users; relatives of the intended users; education experts and social workers.<br>The most common methods used were focus groups (n = 5) and surveys (n = 5), followed by single-person formative interviews (n = 4) and single-person design or prototype testing sessions (n = 4) and advisory group discussions (n = 3) and surveys (n = 3).		
Nu and Bersamin 2016[68]<br>USA	To describe formative research and an ongoing collaborative process to design a multilevel nutrition intervention.<br>Focus groups. Thematic analysis.	This study used qualitative data about the connection between salmon and well-being for a collaborative review (10 focus groups) with a community working group to co-design a nutrition intervention.	10 community representatives with links to the school: Elder, tribal council members, city government, former and current teachers, parents, school and university students, representatives from fishing and business development sectors, and other respected community leaders.	Effectiveness not assessed.	*Co-design*<br>Abstract
Rosso and McGrath, 2016[69]<br>Australia	To report on a pilot project of a sport-based community development program to empower	In collaboration with university staff, local leaders and local stakeholders, 5 location-specific programs were designed for children and youth who	Regional partnership action group (representatives of government agencies, state sport organisations and a local university); community	The pilot shows that disadvantaged CALD communities can be effectively engaged around sport and that participation in	*Co-design*<br>Abstract<br>Discussion

Reference / Country	Aim / Methodology	What is meant by co-production in the development and evaluation of chronic disease prevention interventions?	Who is involved in the co-production of the development and evaluation of chronic disease prevention interventions?	How effective (process and impact measures) are chronic disease prevention programs developed using co-production processes?	Where in the paper is co-production (or other 'co-word') mentioned?
	CALD communities to engage in health promotion through sport.<br>Participatory action research approach.	did not participate in any other forms of organised sport for psychosocial, cultural and economic reasons.	champion from each of five communities.	community sport activities can in turn provide a basic but valuable forum for broader promotion of physical activity and health.	
Standoli et al., 2016[70]<br>Italy, Spain and UK	To describe the co-design approach to developing and evaluating wearable monitoring systems addressed to adolescents.<br>Co-design focus groups.	Focus groups for teenagers (with a focus on community inclusion – all students in a class rather than those with identified risk factors). Testing of devices by a sample of teenagers.	Potential users – teenagers, and researchers.	System reliability tested. Through a co-design activity and approach, user requirements and preferences were addressed.	*Co-design*<br>Abstract<br>Key words<br>Background<br>Methods
Isbell et al., 2015[71]<br>USA	To describe the Texas Special Supplemental Nutrition Program for Women, Infants, and Children (WIC) which sought to engage the WIC staff and community in the implementation of relevant and effective client cantered nutrition education.<br>Descriptive.	Co creation is not defined, but a four-stage process is described: 1. Conceptual stage, 2. Pilot stage, 3. Developmental stage, 4. State-wide implementation.	Staff and community and researchers	Effectiveness not assessed.	*Co creation*<br>Abstract<br>Background<br>Methods
Mackenzie et al., 2015[72]<br>UK (England)	To explore the acceptability and feasibility of a low-cost, co-produced, multi-modal intervention to reduce workplace sitting.<br>Used participative approaches and "brainstorming" techniques.	The development of the intervention included *formative research* (a participative approach with the target audience to engage and promote 'buy-in').	Target population (volunteers from a UK university department).	Small-scale pilot provides encouragement for the acceptability and feasibility of low-cost, multi-modal interventions to reduce workplace sitting in UK settings. Evaluation of this intervention provides useful information to support participatory approaches during intervention development and the potential for more sustainable low-cost interventions.	Co-produce<br>Title<br>Abstract<br>Discussion
